# Combinatorial Therapeutic Approaches with Nanomaterial-Based Photodynamic Cancer Therapy

**DOI:** 10.3390/pharmaceutics14010120

**Published:** 2022-01-04

**Authors:** Yang Hao, Chih Kit Chung, Zhenfeng Yu, Ruben V. Huis in ‘t Veld, Ferry A. Ossendorp, Peter ten Dijke, Luis J. Cruz

**Affiliations:** 1 Translational Nanobiomaterials and Imaging (TNI) Group, Department of Radiology, Leiden University Medical Center, Albinusdreef 2, 2333 ZA Leiden, The Netherlands; y.hao@lumc.nl (Y.H.); c.k.chung@lumc.nl (C.K.C.); z.yu@lumc.nl (Z.Y.); r.v.huis_in_t_veld@lumc.nl (R.V.H.i.‘t.V.); 2 JeNaCell GmbH, Winzerlaer Straße 2, 07745 Jena, Germany; 3 Percuros B.V., Zernikedreef 8, 2333 CL Leiden, The Netherlands; 4 Department of Immunology, Leiden University Medical Center, Albinusdreef 2, 2333 ZA Leiden, The Netherlands; f.a.ossendorp@lumc.nl; 5 Department of Cell and Chemical Biology and Oncode Institute, Leiden University Medical Center, Einthovenweg 20, 2300 RC Leiden, The Netherlands

**Keywords:** cancer photodynamic therapy, drug delivery, combined therapy, cancer vaccines, chemotherapy, radiotherapy, checkpoint inhibitor therapy

## Abstract

Photodynamic therapy (PDT), in which a light source is used in combination with a photosensitizer to induce local cell death, has shown great promise in therapeutically targeting primary tumors with negligible toxicity and minimal invasiveness. However, numerous studies have shown that noninvasive PDT alone is not sufficient to completely ablate tumors in deep tissues, due to its inherent shortcomings. Therefore, depending on the characteristics and type of tumor, PDT can be combined with surgery, radiotherapy, immunomodulators, chemotherapy, and/or targeted therapy, preferably in a patient-tailored manner. Nanoparticles are attractive delivery vehicles that can overcome the shortcomings of traditional photosensitizers, as well as enable the codelivery of multiple therapeutic drugs in a spatiotemporally controlled manner. Nanotechnology-based combination strategies have provided inspiration to improve the anticancer effects of PDT. Here, we briefly introduce the mechanism of PDT and summarize the photosensitizers that have been tested preclinically for various cancer types and clinically approved for cancer treatment. Moreover, we discuss the current challenges facing the combination of PDT and multiple cancer treatment options, and we highlight the opportunities of nanoparticle-based PDT in cancer therapies.

## 1. Introduction

Each year, about 10 million people die of cancer, accounting for about one-sixth of the worldwide mortality, thus causing a high societal and economic burden [1]. Patients in the early cancer stages (stage I/II) can often be efficiently treated by conventional approaches, such as surgery, chemotherapy, and radiation therapy [2]. However, more aggressive stages of cancer are difficult to treat; therefore, new therapeutic options are desired. In photodynamic therapy (PDT), a light source is used in combination with a photosensitizer and oxygen in order to induce cell death. PDT is used most commonly to treat acne and other medical conditions, including psoriasis and age-related macular degeneration [3]. Notably, its application to therapeutically target primary tumors with negligible toxicity and minimal invasiveness has gained great momentum. Patients are administered with a photosensitizer first, which accumulates in tumors. By exposure to specific wavelengths of nonthermal light, the photosensitizer becomes activated from the ground to excited states, thereby providing energy for oxygen to generate reactive oxygen species (ROS), including hydrogen peroxide (H_2_O_2_), superoxide anions (O_2_^−^), and hydroxyl radicals (OH^−^), and singlet oxygen (^1^O_2_) [4]. This destroys the organic constituents of the (tumor) cell structure, triggering apoptosis and necrosis of the cancer cells [5]. Furthermore, PDT can also achieve antitumor effects indirectly, by damaging the tumor vasculature and by activating immune responses [6].

Over the past 30 years, PDT has been tested clinically for different cancer types, especially superficial tumors, such as oropharyngeal cancer, esophageal cancer, and cutaneous carcinoma [7]. Due to the penetration limitations of traditional visible light into tissues, PDT has not been used for the treatment of large tumors that are growing in internal organs to date. The penetration constraints provide a challenge that needs to be overcome. Moreover, to enable PDT treatment of cancer relapses, further optimization and the development of treatment strategies utilizing PDT combined with currently available cancer treatment modalities are needed. Furthermore, a better understanding of the underlying mechanisms of such PDT combined therapies is required.

In this review, we introduce the mechanisms of PDT-mediated tumor ablation and summarize the recent clinical advances and challenges of PDT. Additionally, chemotherapy, targeted therapy, and immunotherapy, among others, have been shown to be excellent combination partners of PDT. In light of the above, we provide a review of these PDT combination strategies and how nanomedicine can help to enhance the anticancer effects of these combinations.

## 2. Photodynamic Therapy

### 2.1. Mechanism of Photodynamic Therapy in Cancer

**Figure 1:**
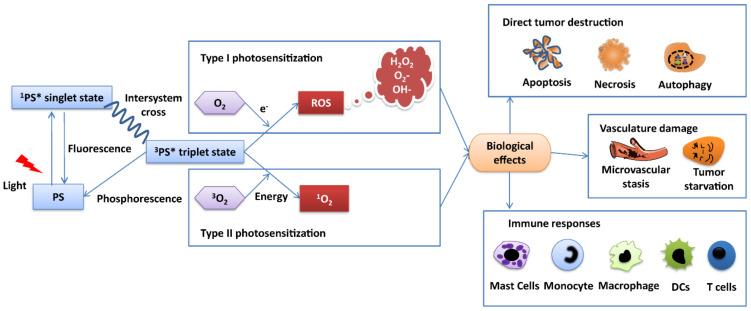
Mechanism of photodynamic therapy in cancer. The antitumor effects of PDT include three main mechanisms: PDT-induced cellular toxicity, vascular destruction, and immune response activation. When exposed to excitation wavelength light, the ground-state photosensitizer moves to a singlet state. In this state, PS can decay by emitting fluorescence, react with biological substrate, or undergo intersystem crossing, thereby being converted into a triplet state with longer life span (microseconds) and parallel spins. Triplet excited PS directly interacts with cell substate to generate toxic reactive oxygen species to directly kill tumor cells by inducing apoptosis, necrosis, or autophagy. PDT also induces tumor vasculature damage and immune responses. Abbreviations in figure: photosensitizer (PS), photosensitizer first excited state (^1^PS*), photosensitizer triplet excited state (^3^PS*), water (H_2_O), triplet oxygen (^3^O_2_), singlet oxygen (^1^O_2_), reactive oxygen species (ROS), hydrogen peroxide (H_2_O_2_), superoxide anions (O_2_^−^), hydroxyl radicals (OH^−^).

Initially, PDT was commonly used to treat nonmalignant diseases (acne and age-related macular degeneration) [8,9,10]. Since the mid-1950s, PDT has been explored as a treatment option in a large variety of preclinical cancer models; when increased specificity and selectivity was achieved in the early 1990s, clinically approval was obtained for cancer treatment [11]. For example, PDT is used to target lung tumors, esophageal cancer, gastric carcinoma, breast cancer, brain tumors, head and neck tumors, colorectal cancers, etc. [12]. PDT is a multistage process, based on three components: a photosensitizer (PS), a light source, and tumor oxygen. It exerts its tumor destruction effects through photochemical and photobiological mechanisms [13] (Figure 1). The PS has negligible cellular toxicity under a lack of light, regardless of the route of administration. An appropriate light dose can provide enough energy for the accumulated PS in the diseased tissue to move into an excited state from the ground state, leading to the production of free radicals and ROS. Depending on the nature of this reaction, such photosensitized processes are defined as Type I and Type II. During the Type I process, triplet excited PS directly interacts with the cell substate to generate free radicals (e.g., hydroxyl radicals, superoxide anion, and hydrogen peroxide) through a hydrogen atom (electron) transfer. These radicals can further interact with oxygen to produce toxic reactive oxygen species. A Type II process, however, produces highly reactive singlet oxygen (^1^O_2_) via oxygen (^3^O_2_) through electron transfer. These reactive species are highly cytotoxic and directly kill tumor cells by inducing apoptosis, necrosis, or autophagy [14]. However, the kind of cell death induced by the PDT treatment depends on the characteristics of the PS (e.g., intracellular location and activation wavelength), cell type, and PDT dose (including PS concentration and total light fluence) [15]. Moreover, the destruction of tumor cell results in the production of new tumor-derived antigens and the increased expression of stress proteins. These PDT-killed tumor pieces are phagocytosed by macrophages and lead to acute inflammation, leukocyte infiltration, and maturation activation of dendritic cells [6]. PDT also reduces tumor volume indirectly by inducing microvascular shutdown and vessel leakage. This event can lead to nutrient starvation and hypoxia [16]. In general, the function of these mechanisms is cooperative, but which particular mechanism is dominant in PDT’s tumor-controlling effects is still unclear and requires further study.

### 2.2. Generations of PS

**Figure 2:**
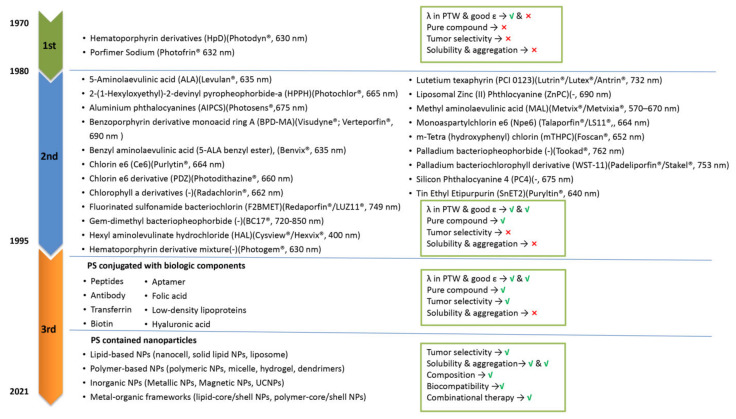
Different generations of PSs widely used in various cancer cell types. Currently developed PSs can be divided into first-generation PSs, second-generation PSs, and third-generation PSs. The description is provided as follows: ● chemical name (abbreviation)(trade name is indicated with^®^, and excitation wavelength is indicated in “nm“ during clinical PDT procedure). If information is not available, this is indicated with (-). λ in PTW represents the typical wavelength at which absorption of photosensitizer occurs to penetrate into tissues (PTW, λ > 600 nm), and ε represents the absorption rate at PTW.

The advantages of PDT are its low systemic toxicity, its minimal invasiveness, and its targeting opportunities. The therapeutic efficacy of PDT depends on the properties of light, availability of sufficient tissue oxygen, and PS characteristics (uptake and localization). However, further studies are needed for PDT to achieve a better therapeutic effect with fewer shortcomings. For example, a superficial irradiation approach for noninvasive PDT has the limitation of tumor tissue penetration. However, this can be improved by coupling PDT to optical fibers or intraluminal/interstitial settled multi light sources [17]. Moreover, hypoxia, the major barrier of PDT efficiency and the main reason for PDT resistance, can be counteracted by PS dosimetry [18,19,20,21]. In addition to the improvement of irradiation light equipment and optimization of oxygen ratio in the tumor, there is a need for further optimization of PSs. So far, PSs can be categorized into three generations [22]. First-generation PSs were developed in the 1970s and include hematoporphyrin derivatives (HpD) and its purified form, as well as Photofrin (trade name of porfimer sodium) [23] (Figure 2). Whereas certain antitumor effects of Photofrin have been reported for several types of cancer (brain, lung, skin, gastric, etc.) in clinical tests [24], some drawbacks (e.g., complex composition, weak absorption at 630 nm) and obvious side-effects (light-dependent skin sensitivity caused by the high PS dose that is needed to achieve therapeutic effects) of first-generation PSs limited their clinical application [7,24,25]. These shortcomings triggered the development of second-generation PSs (Figure 2). The second generation was still based on porphyrin and chlorin structures, but their purity and synthesis were improved. Furthermore, second-generation PSs had a longer light activation wavelength and shorter half-life [26]. Examples include 5-aminolaevulinic acid (ALA), temoporfin (Foscan^®^), palladium bacteriopheophorbide (Tookad^®^), tin etiopurpurin (Purlytin^®^), and benzoporphyrin derivative monoacid ring A (BPD-MA; Visudyne^®^; Verteporfin^®^). 5-ALA is a key precursor to the synthesis of heme. On the basis of this characteristic, 5-ALA is used as a prodrug for PDT by producing PPIX (photosensitizer), the immediate precursor of heme. ALA derivatives such as methyl, benzyl, and hexyl ALA ester have also been approved for use in cancer diagnosis and treatment [27]. As we discussed in Section 2.1., PDT can impair vascular structures or induce microvascular stasis, depending on the PS type and protocols used. For example, vascular targeted PDT with BPD-MA (VP, Verteporfin^®^) can effectively induce endothelial cell injury to cause vascular damage [28]. Another example is Radachlorin^®^-mediated PDT. In a typical protocol, after 4 h intravenous injection of Radachlorin^®^ into tumors, irradiation is provided at 100 mW/cm^2^ for a total light dose of 20 J/cm^2^ using a 662 nm laser. Five days after PDT treatment, intravital imaging revealed a disrupted tumor vasculature [29]. The major difference between the first- and second-generation PSs is the diffusion rate of the PDT-generated singlet oxygen and ROS caused by their subcellular uptake in organelles such as lysosomes, nuclear envelope, and mitochondria [30]. The diffusion rate of the PDT-generated ROS caused by the particular uptake of PSs leads to a difference in PDT sensitivity and PDT-induced cell death type, because of the short ROS half-life time [30,31]. PSs localized mitochondrially and in other organelles induced more ROS generation and induced significantly higher photodamage efficacy than PSs taken up by lysosomes [32].

Despite the improved therapeutic effect of second-generation PSs, the complex tumor microenvironment (especially PDT-enhanced degree of hypoxia) and the glutathione (GSH) depletion effects on ROS weaken the toxic efficiency of PDT-generated ROS [33]. Moreover, the hydrophilicity, tumor selectivity, and body clearance rate of PSs were far from optimal. For example, Foscan^®^, which needs to be injected in a painful way in a polyethylene glycol, ethanol, and water mixture, demonstrated no significant difference in fluorescence between tumor tissue and its surrounding tissues in a rat breast cancer model [34]. Such challenges have endorsed research on the further optimization of PSs to the third generation of compounds [35] (Figure 2). The selectivity problem of PSs for tumor tissue over healthy tissue has been addressed by the covalent binding of PSs to ligands, such as folate, transferrin, peptides, and antibodies. Such PS conjugation enabled more selective recognition and internalization by tumor cells, thus minimizing damage to healthy cells. As certain receptor sites on tumor cell surfaces, such as biotin, androgen, and glucose receptors, are highly expressed on tumor cells, the conjugated targets for PSs enable more selectivity in cancer cell targeting [36].

An alternative approach for optimization would be to increase the efficiency and selectivity of the PS delivery system [37]. An emerging solution in this line comprises the use of nanoparticles (NPs; 1–100 nm). Owing to their enhanced permeability and retention (EPR) effect, as well as subcellular size, NPs have been shown to support PSs, in order to penetrate deeper into tissue and preferably accumulate in tumors [38]. These NPs can increase the PS stability, reduce its degradation before it accumulates in tumor cells, and improve the hydrophobic PS solubility by increasing its aggregation in an aqueous environment. Additionally, modifying the surface of the NP with targeting components also offers more opportunities for PSs to be delivered more specifically in diseased tissues [39]. As a result of enhanced PS delivery to tumor cells, a larger concentration is available to harness stronger PDT effects, without inducing excessive off-target systemic side-effects [40,41].

## 3. Clinical Development of PDT Combined Therapy in Cancer

**Figure 3:**
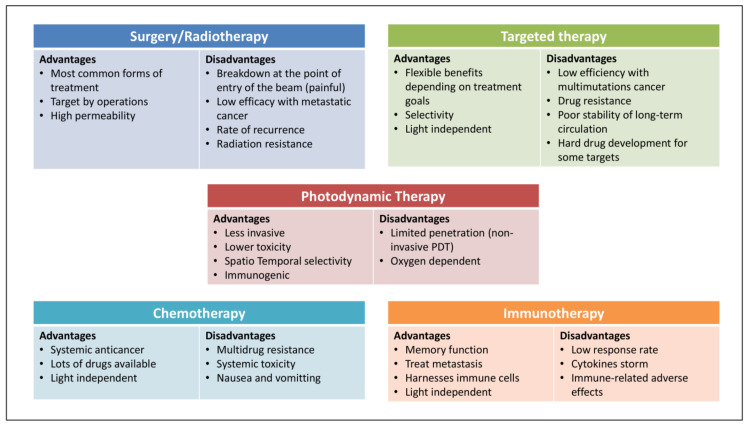
Summary of the advantages and disadvantages of major cancer therapies. PDT-based combination therapies for the treatment of cancer integrate the advantages and bypass the disadvantages of monotherapies, including surgery, radiotherapy, targeted therapy, immunotherapy, and other combined strategies.

As illustrated above, important for clinical approval has been that PDT is noninvasive and toxic, spatiotemporally selective, and not very immunogenic. However, the therapeutic efficacy of PDT alone against several deep or hypoxic solid tumors is limited due to its inherent drawbacks and the clinical challenges (metastasis, recurrence, and resistance) of cancer therapy [42,43]. The mechanisms that contribute to PDT resistance might be changed in drug uptake and efflux rates of PSs, activation of abnormal cell signaling pathway activation, and hypoxia after PDT. However, two-thirds of the reports showed no cross-resistance to chemotherapy-, radiotherapy-, and hyperthermia-resistant cells in PDT-resistant cells [44]. From this perspective, by combining PDT with other current cancer modalities, one may be able to exploit the strengths and bypass the weaknesses of different therapies (Figure 3). As presented in the subsequent sections, this approach has great promise and can lead to additive (or even synergistic) therapeutic effects [45]. Consistent with this notion, PDT-combined strategies have gradually entered into clinical trials for the treatment of basal cell carcinoma, non-small-cell lung cancer, and other types of cancer. In particular, its combination with surgery, radiotherapy, and chemotherapy has been investigated (clinically trailed data was collected on 20 August 2021 from resource: http://clinicaltrials.gov; Table 1). Further efforts are needed to discover new PSs, specifically for deeper located cancers, and to optimize PS-mediated PDT in various tumor types.

### 3.1. PDT Combined with Surgery

PDT has been frequently used in conjunction with surgery in clinical cancer trials (Table 1) due to the image-guided effect (NCT03638622) and increased anticancer therapeutic effect [46]. A phase I clinical trial (NCT00470496) of intraoperative PDT combined with surgery in the treatment of primary or recurrent head and neck cancer showed an improved cure rate, by allowing for larger tumor-free margins while preserving normal structures. A clinical study of surgical PDT underscored that there was no relapse (follow-up of 0.6–5 years) in basal cell carcinoma (BCC) patient tissues after combined treatment. Moreover, transmission electron microscopy analysis of tumor tissues indicated fewer side-effects in patients after treatment [47]. In addition, when PDT was combined with surgery, the tumoral depth showed less limitation in skin cancer patients. Post-surgical PDT improved not only the efficacy of tumor thickness reduction and the survival rate in both squamous cell carcinoma and basal cell carcinoma patients [48], but also the recovery rate and appearance satisfaction by reducing the excision range of the tumor lesions [49]. In addition to skin cancer, the effectivity and safety of neoadjuvant PDT to surgery has been shown in preclinical trials for the treatment of non-small-cell lung cancer [50], breast cancer (extramammary Paget’s disease; EMPD) [51], and mesothelioma [52].

However, research has shown that surgery can induce the production of inflammatory mediators such as IL-6; these inflammatory cytokines can lower the effects of PDT by changing the tumor microenvironment and affecting the immune system [53]. This effect can be extenuated to improve the survival rate by increasing the time interval between surgery and PDT to 6 weeks [54]. Thus, the antitumor effect by combining PDT and surgery is worth further exploration in subsequent clinical trials.

### 3.2. PDT Combined with Radiotherapy

PDT combined with radiotherapy (RT) is the second major combination approach in clinical trials (Table 1). PDT-RT has superior therapeutic efficacy over PDT or radiotherapy alone. Decades ago, Calzavara et al. noticed that adjuvant radiation therapy after PDT in esophageal cancer served as an effective treatment for patients [55]. For further confirmation of this observation, an incomplete survey in Japan, from January 1986 to March 1992, showed that PDT and external beam radiation therapy had almost 100% curative power for roentgenologically occult lung cancer (except for noncancerous lethal) [56]. Not accidentally, other clinical data have shown that the combination of PDT and brachytherapy (high dose) was safe and excellent for lung cancer, with no recurrence, no severe complications for 28 patients, and two complications in six patients with metastases (32 patients in total) [57]. Furthermore, PDT followed by ionizing radiation has been reported to be a more safe and well-tolerated palliative treatment to prevent and alleviate suffering, thereby improving the life quality of patients facing life-threatening advanced esophageal cancer [58]. Studies have also demonstrated that ALA-PDT together with deeply penetrated holmium or carbon dioxide lasers had curative effects on patients with extramammary Paget’s disease (EMPD), which is a rare and slow-growing intraepithelial neoplasm [59]. Further studies have demonstrated the safety of this combination in EMPD treatment, with fewer side-effects such as refractory ulcers of ionizing radiation [60,61]. Although survival rates after RT can be high in several cancer types, including early-stage larynx cancer and non-small-cell lung cancer, unfortunately, in some other cancers (glioblastomas and sarcomas), there are tumor recurrences because of hypoxia, surviving cell repopulation during RT, and intrinsic cell radioresistance [62]. When PDT is combined with RT, the RT resistance does not influence the efficacy of PDT. Thus, the treatment sequence can be reversed to start with radiotherapy, followed by PDT [63]. To this point, in a phase I study of PDT as an adjuvant treatment for esophageal cancer, the optimum laser fluence rate of PDT was first determined using talaporfin sodium and a diode laser for patients with local failure after chemoradiotherapy or RT [64]. Thereafter, a multicenter phase II study demonstrated the efficacy of this strategy, with an 88.5% local complete response for local RT failure esophageal cancer patients [65].

### 3.3. PDT Combined with Chemotherapy

The clinical trials of PDT plus chemotherapy are currently based on first-generation PSs (porfimer sodium, Photofrin^®^). PDT in combination with standard chemotherapy has been studied in NCT01770132, NCT02082522, NCT00869635, and NCT02662504. Moreover, the possibilities of combination with gemcitabine hydrochloride, S-1, cisplatin, and pemetrexed have been explored. A phase II study (NCT00869635) of PDT combined with systemic S-1 chemotherapy for cholangiocarcinoma showed good tolerance and improved efficacy, with a higher 1 year survival rate (76.2% vs. 32%) and prolonged overall survival (median 10 months vs. 2 months), compared with patients treated with PDT alone [66].

## 4. Nanomedicine-Based Combination Therapy Strategies

**Figure 4:**
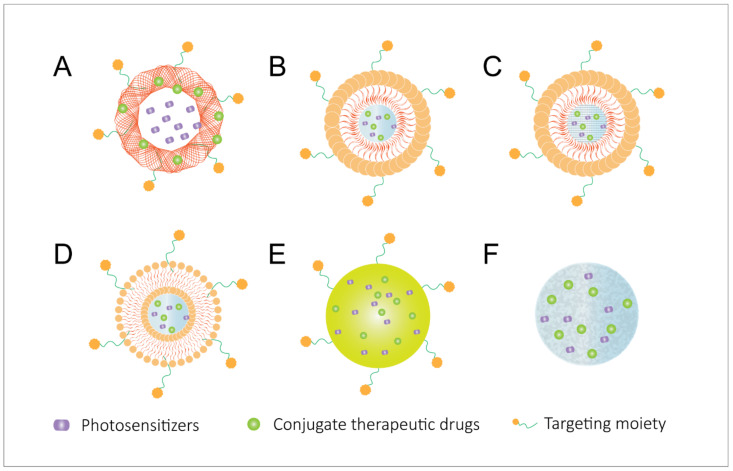
Most used codelivery systems for combined therapies with PDT in cancer, including (**A**) polymeric nanoparticles (PNPs), (**B**) nanostructured lipid carriers (NLCs), (**C**) solid lipid nanoparticles (SLNs), (**D**) liposomes, (**E**) gold nanoparticles (AuNPs), and (**F**) hydrogels.

A few cancer types respond well to traditional methods such as surgery, radiotherapy, and PDT. Unfortunately, several solid tumors fail to respond due to therapy resistance, metastasis to distant organs, and induced recurrence problems in cancer patients [67]. Common mechanisms of metastasis include genomic instability, epigenetic modifications, epithelial-to-mesenchymal phenotype transition (EMT), remodeling the extracellular matrix, blood supply system, immune evasion microenvironment, and metastatic sites, among others [68,69,70,71,72]. Metastasis and therapy resistance may be addressed by the use of nanocarriers to improve the therapeutic index [73]. Employing specifically adapted nanoparticles for PDT-based combined therapy provides a promising platform for codelivery of multiple drugs (action in different modes) and PSs, with the advantages of minimizing potential toxicity in healthy tissues, improving drug efficacy, and excellent physicochemical properties [74]. Of note, a nanotechnology-based PDT combination displayed potential in preclinical studies by incorporating the features of diagnosis, therapy, and imaging. In this sense, highly encouraging results have been obtained through the combination of PDT with organic delivery systems (e.g., hydrogels, liposomes, and polymeric nanomaterials) and inorganic nanomaterials (e.g., metallic and silica NPs) (Figure 4).

The utilization of nanoparticles as delivery systems for PDT combinations can function in four different ways: (1) drug protection—protecting therapeutic cargos (e.g., drugs, antigens, and adjuvants) and PSs from degradation during blood circulation and prolonging their retention period; (2) tumor targeting—modifying the surface of the NPs with components that can interact with overexpressed molecules on tumor cell surface, thereby decreasing the nonspecific uptake of NPs to healthy cells and enhancing the accumulation of the NPs in tumors; (3) tumor normalization—overcoming PDT-enhanced hypoxia in the tumor microenvironment by intracellular oxygen supply by NPs or drug loading of hypoxia-activated prodrugs, such as tirapazamine (TPZ), apaziquone (EQ4), and banoxantrone (AQ4N), or overcoming the neutralization of PDT-generated ROS by high glutathione (GSH) in the tumor microenvironment through GSH-activated NPs or chemicals with the ability of intracellular GSH depletion; (4) medical imaging—protecting aggregation-caused quenching (ACQ) of PSs and providing opportunities to integrate multi-imaging modalities.

### 4.1. Nanoparticle-Based PDT Plus Surgery

PDT has been found to act as an effective adjuvant therapy in image-guided cancer surgery, especially in prostate cancer [75,76]. The PSs can improve the visualization of tumor margins and metastatic lymph node drainage, due to their fluorescent nature. Subsequent PDT treatment further ablates the remaining tumor tissues during surgical resection, thereby reducing tumor recurrence and significantly extending survival. Utilization of nanoparticles in PDT synergized with surgery to overcome ACQ of PSs during their introduction and increased the uptake and retention time of the PSs for imaging guidance of the surgery [77]. For example, PS-loaded gold nanoparticles (AuNPs) [78], up-conversion nanoparticles (UCNPs) [79,80], and conjugated polymer (CP) nanoparticles [81] have shown promising potential to be used in imaging-guided surgery and PDT. A novel multimodal porphyrin lipoprotein-mimicking nanoparticle labeled with copper-64 (PLP) intrinsically integrates diagnosis (positron emission tomography (PET) imaging and fluorescence imaging) and PDT treatment in this platform [82].

### 4.2. Nanoparticle-Based PDT Plus Radiotherapy

Studies have demonstrated that nanoparticle-based PDT plus radiotherapy improved antitumor effects by improving the absorption efficiency and stability of agents (PSs and radio agents), thus reducing the side-effects of PDT and RT to healthy organs. For example, Wang et al. set up self-assembling nanoparticles (Ce6-R9-^125^I-RGD-MNPs) of PS and radiotherapeutic peptides, which showed a better tumor-inhibitory effect compared to single therapy but with minimal toxic effects to normal tissues in Hela tumor-bearing mice [83]. A nanoparticle consisting of hafnium (radiosensitizer) and tetrakis (4-carboxyphenyl) porphyrin (TCPP, as PS) has been found to have a higher capacity to destroy tumor cells than single RT or PDT in a 4T1 murine breast cancer model because of longer tumor retention time [84]. This combination strategy has the potential to further improve the PDT efficiency in deep tumors due to the penetration ability of ionizing irradiation (X-ray) to the tumors. Liu et al. demonstrated that dibenzocyclooctyne (DBCO)-modified Hf-AIE coordination polymer nanoparticles (CPNs) has good biosafety, using hematoxylin and eosin (H&E) staining images of tumors. It can also greatly inhibit a 4T1 murine breast cancer model due to increased CPN tumor accumulation and prolonged retention time. Furthermore, CPNs have been found to have an improved anticancer effect against a deep tumor model (42.5% tumor growth suppression) [85].

In addition to the increased single-therapy threshold by the delivery system, PSs (e.g., Photofrin II and hematoporphyrin dimethyl ether; HPde) [86,87] and high-Z nanomaterials (e.g., gold nanoparticles (AuNPs), MoS_2_/Bi_2_S_3_ nanosheets, and CuS nanoparticles) can act as specific radiosensitizers, in order to obtain an optimized anticancer effect. From this perspective, researchers have developed hyaluronic acid-modified Au nanocages (AuNPs-HA) integrating photoacoustic (PA) imaging, RT, and PDT at the same time. This multiple functional nanoplatform itself works as both radiosensitizer and PS, leading to better tumor growth suppression than each therapy alone in a 4T1 murine model. Additionally, the PA imaging-guided approach enabled more precise identification of the tumor location and size [88].

At a certain point, the improved absorption difference between healthy and tumor tissues by nanocarriers may minimize the resistance problem of RT. Geoffrey et al. generated MC540-SAO:Eu@mSiO_2_ nanoparticles (MC540: a PS; SAO: Eu, a scintillator that converts X-ray photons to visible photons). These nanoparticles were used in combined PDT and RT. MC540-SAO:Eu@mSiO_2_ NPs enhanced antitumor growth effects and reduced clonogenicity of RT-resistant cancer cells in an H1299 mouse model without detectable systematic toxicities [89]. Further studies have demonstrated that nanoparticle-based PDT-RT can have systemic synergistic antitumor effects through an enhanced apoptosis rate by targeting different cellular components (e.g., cell membrane and DNA) leading to facilitated ROS diffusion, the release of damage-associated molecular patterns (DAMPs; molecules released from damaged or dying tumor cells can induce innate immune responses), and antigen expression [90]. These mechanisms have supported studies to explore the strategy of combining PDT-RT with immune checkpoint inhibitors in order to stimulate an activated immune system, especially CD4^+^ and CD8^+^ T cells. Immunotherapy PDT combinations are discussed in detail in Section 4.4. When combined with anti-PDL1, PDT-RT treatment results have shown tumor growth inhibition indices of primary and distant tumors as 99.5% and 98.0%, respectively, for CT26, and 94.7% and 92.2%, respectively, for SCC VII tumor models [91]. When further combined with an indoleamine 2,3-dioxygenase (IDO) inhibitor, which acts on tumor cells by enhancing antigen recognition, PDT-RT nanoparticles regressed both primary-treated tumors and distant untreated tumors by forming an in situ vaccine in a CT26 colon-rectal cancer murine model [92].

### 4.3. Nanoparticle-Based PDT Plus Chemotherapy

**Figure 5:**
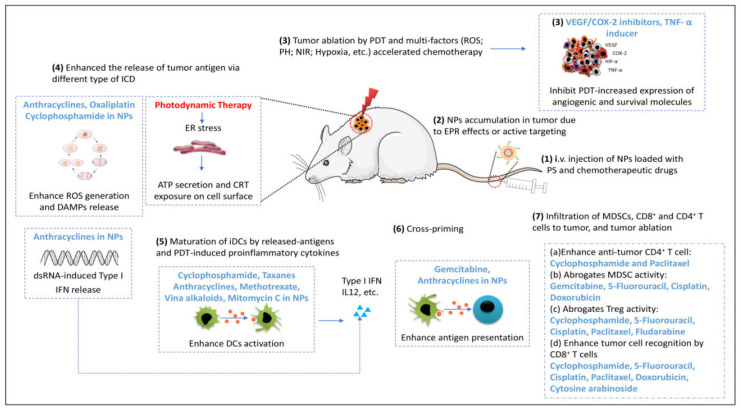
The mechanisms of NP-mediated chemo-photodynamic therapy enhance monotherapy indices and synergistically trigger robust antitumor immune responses for anti-primary and metastatic tumors: (1) intravenous injection of PSs and chemotherapeutic agents loaded with nanoparticles; (2) enhanced NP accumulation in tumor due to the tumor-targeting capability of NPs (EPR effects and targeting motif modification on NP surface); (3) primary tumor ablation by enhanced PDT and chemotherapy. PDT-caused vasculature rupture induced surviving tumor cells to produce more protumor factors in the tumor microenvironment. Antiangiogenetic mediators, such as VEGF and COX-2 inhibitors, help PDT to achieve more powerful tumor destruction and a lower recurrence or metastasis rate, by blocking tumor angiogenetic activity molecules or their receptors; (4) PDT and chemotherapy (anthracyclines, cyclophosphamide, and oxaliplatin) of the primary tumor to induce higher ICD levels and the release of tumor-associated antigens. Anthracyclines also induce dsRNA release from dead tumor cells, which can activate tumor-specific CD8^+^ T cells by binding to Toll-like receptor-3 and inducing type I interferon production; (5) DC maturation and antigen presentation are enhanced by PDT-generated antigens, proinflammatory cytokines, and chemotherapeutic agents; (6) cross-priming in tumor lymph node; (7) Chemotherapeutic agents in NPs can improve PDT-induced immune responses by modulating the activity of immune cell subsets and by promoting tumor cell death.

Chemotherapy (CT) is the main antitumor treatment modality, which works by inhibiting the process of cell growth and cell division by binding to tumor cell DNA. As shown in Figure 5, co-loading PSs and antitumor chemo drugs into the same delivery system can enhance the effects of single-therapy approaches. Some small-molecular inhibitors co-encapsulated with PSs can help PDT to greatly destroy primary tumors with lower recurrence or metastasis rates. Blocking angiogenic activity molecules or their receptors against tumors possibly upregulates expression of vascular endothelial growth (VEGF) and cyclooxygenase (COX)-2 during PDT [93,94]. Additionally, they can achieve a spatial cooperation anticancer effect via synergistic effects through enhancing immune responses by increasing immunogenic cell death (ICD) levels (ICD is defined by chronic exposure of DAMPs), type I interferon (IFN) secretion, and modulating immune cell subset activities [95]. Furthermore, the decreased effective dosage of therapeutic agents in a codelivery system can result in a reduction in side-effects while providing the potential of reducing multidrug resistance (MDR) [96]. Furthermore, some specific targeting ligands can be modified to the surface of NPs in order to enhance the tumor accumulation of drugs and decrease the severe side-effects of chemotherapy drugs due to their non-specificity, thus enhancing antitumor efficiency. For example, folic acid (FA), hyaluronic acid (HA), biotin, and antibodies have been utilized on the surface of dual drug-loaded nanosystems as active targeting ligands [97]. Yumin et al. conjugated RGD peptides to pH-sensitive polyethylene glycol (PEG) nanoparticles containing Ce6 as a PS and doxorubicin (DOX) for chemotherapy. These nanoparticles have a highly cytotoxic effect in vitro, due to improved cellular uptake. The NPs significantly enhanced the antitumor effect in an MDA-MB-231 tumor-bearing mouse model, with lower cardiotoxicity of DOX because of the superior tumor targeting and retention ability of NPs [98].

#### 4.3.1. Organic Nanoparticle-Based PDT Plus Chemotherapy

Organic NPs have attracted attention in the field of PDT plus chemotherapy (Table 2), due to their biosafety and biocompatibility profiles. Several well-developed structures have been widely used for PS and chemo-drug codelivery, including polymeric NPs, micelles, liposomes, hydrogels, and dendrimers.

Polymeric NPs used for PDT combination consist of naturally occurring (e.g., alginate, chitosan, and collagen) or synthetic polymer (e.g., polylactic acid (PLA), polyglycolic acid (PGA), or their copolymers, such as polyester (PLGA) and polyethylene glycol (PEG)), which can be hydrolyzed enzymatically into nontoxic byproducts in metabolic environments [99]. For example, highly tumorigenic cancer stem cells (CSCs) in tumors are one of the main reasons for chemotherapy resistance. Elisa et al. reported the self-assembly of hyaluronic acid (HA)-coated polymeric nanoparticles using PEI–PLGA, docetaxel (DTX), and *meso*-tetraphenyl chlorine disulfonate (TPCS2a). After intravenous injection of NPs, HA@DTX/TPCS2a-NPs accumulated more in monolayers and mammosphere cultures enriched in CSCs (CD44^high^/CD^24^ low population) and elicited superior efficacy over monotherapies in reducing the self-renewal capacity. These nanomaterials showed great potential to overcome CSC-induced chemo-drug resistance and metastases [100].

Xue-Liang et al. designed and prepared macrophage cell membrane (CM)-coated liposomes to co-deliver nano-platinum (Pt) and verteporfin (VP). This lipid-based nano-Pt/VP@MLipo significantly inhibited tumor cell viability in 4T1 cells and a 3D 4T1 spheroid model. In vivo results showed that there was ~90% 4T1 tumor inhibition in the same period and extended mice survival (median survival 43 days), with no lung metastasis, compared to other treatments [101]. Another study reported hybrid PLGA/lipid-PEG NPs containing indocyanine green (ICG) and TPZ. Via NIR irradiation, ICG-based PDT directly kills the tumor by ROS generation, while consumption of oxygen during the PDT process can promote a degree of hypoxia at the tumor site(s), which may greatly activate the cytotoxicity of the hypoxia-activated TPZ through a cascade process. Furthermore, they demonstrated that this combination by PLGA/lipid-NPs had a synergistic inhibitory effect on primary tumor growth and metastasis-associated with enhanced the necrotic area (~95%) compared to the control group (~30%), via H&E analysis of tumor sections [102]. Micelles and hydrogels have also been studied as codelivery carriers to target tumor cells, due to their enhanced EPR effects. A thermal-responsive hydrogel based on a PCL–PTSUO–PEG copolymer designed by Zhongming et al. had the advantages of local targeting and sustained release. This in situ formed hydrogel encapsulated with DOX and ZnPC showed excellent cell-inhibitory effects in 5637 cells with a cell viability of 18.5% (4.8-fold that of free ZnPC-PDT), due to increased ROS generation. Enhanced ability of tumor control has been observed in a nude mice xenograft bearing 5637 cells [103]. Hua et al. found that self-assembled polyethyleneimine–nitroimidazole (PEI–NI) micelles provided a promising codelivery system for DOX and Ce6. This micelle-based combination of PDT and chemotherapy improved the therapeutic ratio of these modalities, by enhancing the stability and biocompatibility of agents, as well as dual trigger-induced highly cancer-selective drug release [104]. Taken together, organic nanoparticles provide effective delivery systems for PDT in combination with chemotherapy and are being currently studied in both preclinical tests and clinical trials.

**Table 2:** Preclinical studies on organic nanoparticles for codelivery in PDT plus chemotherapy.

PS	Chemo Drugs	Delivery System	Specific Function of Delivery System	Cancer Models	Therapeutic Outcomes of Combination	Ref
Polymeric Nanoparticles						
Ce6	DOX	RGD–PEG–DOX nanoparticles	pH-responsive; tumor targeting by RGD peptide	MDA-MB-231 cells, MCF-7 cells; MDA-MB-231 tumor-bearing mouse model	High cytotoxicity effect in vitro due to improved cellular uptake; significantly enhanced antitumor effect with lower cardiotoxicity of DOX, according to the pathological analysis	[98]
Ce6	Curcumin	Crosslinked polyphosphazene nanoparticles (FHCPCe NPs)	PH/redox dual-stimuli-responsive; dual-modal imaging (fluorescent imaging (FL) and computed tomography (CT))	HeLa xenograft cervical cancer mouse model	Synergistic antitumor activity both in vitro and in vivo	[105]
Ce6	DOX	MnO_2_-loaded PCLA–PEG–PCLA NPs (CDM NPs)	Intratumoral self-sufficiency of O_2_; trimodal imaging (FL, PA, MRI)	MCF-7 xenograft human breast tumors	Enhanced tumor growth inhibition and the inhibition ratio (IR) calculated by tumor weight was 92.35%, with no appreciable impact on body weight or the major organs in mice	[106]
HPPH	Camptothecin (CPT)	Polymeric nanoparticles	ROS-responsive; dual-imaging (PA and FL)	Nude mice bearing CT26 colorectal cancer	Effectively inhibit tumor proliferation and growth in vitro and in vivo	[107]
TPPS2a	DOX	Copolymer nanoparticles	O_2_-evolving and ROS-activable; tumor targeting by F7 peptide	MCF-7/ADR tumor-bearing mice	Enhanced cell killing effects in vitro; prolonged survival time of combined therapy to 41 days, compared to NP-based PDT (32 days) and free DOX (25 days).	[108]
TPCS2a	DTX	Polymeric nanoparticles (HA@DTX/TPCS2a-NPs)	Tumor targeting ability	CD44^high^ MDA-MB-231 and the CD44^low^ MCF-7 cells; mammosphere	Enhanced killing CSCs effects in vitro by 2D and 3D assay	[100]
TPCS2a	CPT	Double-layered polymeric nanoparticles	Tumor targeting due to HA	DTX-sensitive (HeLa-P, MDA-MB-231) and DTX-resistant (HeLa-R) cancer cells	Synergistic antitumor activity in vitro and reduced DTX dose in NPs by ~2.6- and 10.7-fold in HeLa-P and MDA-MB-231, respectively; reduced DTX doses in NPs by more than 100 times in DTX-resistant HeLa-R cells	[109]
Polymer PFV materials	Prodrug BDOX	DSPE–PEG–iRGD–PFV–BDOX conjugated polymer NPs	Tumor targeting by iRGD peptide; ROS-responsive	PC-3 human prostate cancer cells	Enhanced cancer cell killing effects in vitro due to enhanced tumor cell targeting and uptake	[110]
ICG	Oxaliplatin (OXP)	PLGA–PFP–OXP–ICG NPs	Photoacoustic and ultrasonic imaging	ID8 ovarian tumor mouse model	Improved antitumor effects on cancer cell due to enhanced DAMPs expression	[111]
IR780	DOX	Amphiphilic nanoparticles (F-IR780–PEG)	Intratumorally self-sufficiency of O_2_; NIR-responsive; high oxygen capacity	Nude mice bearing MCF-7 human breast cancer	Remarkable therapeutic efficacy in killing tumor cells and destroying solid tumor	[112]
Hematoporphyrin (HP)	DOX	PEG-modified hematoporphyrin (HPP)-based NPs (HPPD)	Enhanced drug release at pH 5.8, along with laser radiation	MCF-7 human breast cancer cells and MHCC-97H human hepatoma cancer cells; nude mice bearing ADR/MCF-7 human breast tumors	A 12-fold decreased IC_50_ value due to improved drug penetration, resulting in promoted apoptosis in vitro; compared to free Dox, which failed to constrain tumor growth, combined therapy had efficient drug-resistant tumor ablation to an undetectable level in 2 weeks without inducing myocardial injury	[113]
Protoporphyrin (Por)	Epirubicin (EPI)	EPI-loaded cRGD–PEG–PH–PCL–Por	pH sensitivity; tumor targeting due to cRGD	CT26 murine colorectal tumor mouse model	Higher anticancer effectiveness, both in vitro with an IC_50_ = 0.47 μg/mL and in vivo, than that of free EPI	[114]
5,10,15,20-Tetraphenylchlorin (TPC)	PTX dimer (PTX2-TK)	RBC-membrane-coated (TPC–PTX2–TK–PEG) NPs	Prolonged blood circulation and improved tumor accumulation by coating RBC membrane	Nude mice bearing HeLa human cervical carcinoma	Enhances anticancer therapeutic activity; reduces systematic toxicity due to light-triggered drug release, as certificated by H&E staining and serum biochemical analysis of main organs	[115]
NPs	SN38	Multifunctional SN38-conjugated polymeric nanosystem (FA-PDA@PZM/SN38@BSA-MnO_2_)	Intratumoral self-sufficiency of O_2_; MRI imaging	Eca-109-esophageal tumor-bearing mice	Superior antitumor efficacy in Eca-109 tumor-bearing mice with low gastrointestinal toxicity and myelosuppression	[116]
Pyrolipid	Pt	Polymer-based core–shell nanoparticles	Drug release in a triggered manner	Human head and neck cancer SQ20B xenograft murine model	Superior potency and efficacy in tumor regression (83% reduction in tumor volume) at low drug doses in a cisplatin-resistant cancer model	[117]
ZnPc	DTX	Biodegradable core–shell nanoassemblies	Biodegradability and biosafety	HeLa cells, nude mice bearing A375 human amelanotic melanoma	Improved tumor growth-inhibitory effects compared to single therapy	[118]
Lipid-based NPs						
Photosan-2	Cisplatin (CDDP)	Lipid platinum-chloride nanoparticles (LPC NPs)	-	Nude mice bearing SAS squamous cell carcinoma	Significantly enhanced the therapeutic outcome in tumor volume reduction, compared to single therapies (~110.8% tumor growth inhibition); reduced the tumor growth rate	[119]
porphyrin	PTX	Porphyrin–lipid nanoemulsions	Imaging ability	KB xenografts tumor-bearing nude mice	Fourfold reduced PTX (1.8 mg/kg) dose in combined therapy with a superior antitumor effect, compared to single PTX therapy (7.2 mg/kg), resulting in reduced side-effects associated with chemotherapy	[120]
VP	Nano-Pt	Nano-Pt/VP@MLipo	Intratumoral self-sufficiency of O_2_	4T1 breast tumor mouse model	Significantly inhibited tumor cell viability in vitro (2D and 3D model); enhanced tumor inhibition and extended mice survival time with no lung metastasis, compared to monotherapies	[101]
ICG	TPZ	Hybrid PLGA/lipid-PEG NPs	Tumor targeting by RGD peptide; improved penetration	3D tumor spheroids and orthotopic 4T1 breast tumor model	Synergistic cell-killing effect in vitro and effective primary tumor growth and metastasis inhibition; enhanced necrosis (~95% necrotic area) compared to control group (~30%), by analysis of the H&E tumor sections	[102]
Hydrogel						
ZnPc	DOX	Polymer hydrogel	Thermosensitive	Nude mice bearing 5637 human bladder tumors	Excellent cell-inhibitory effects in vitro, with cell viability of 18.5%, which is attributed to a high level of ROS generation (4.8-fold free ZnPC); slightly higher increased survival rate compared to chemo and PDT single groups	[103]
Micelles						
Mitoxantrone (MX)	MX	PEGylated UCNP (UPG) micelles	Tumor targeting by grafting with an anti-EpCAM antibody; dual-modality MR/UCL imaging	BEL-7404 liver carcinoma mouse model	94.4% cell death in vitro for combined therapy, compared to 67.6% for chemo only, which was attributed to the physicochemical property of micelles; remarkable antitumor effect with final tumor volume: 235.5 ± 87.4 mm^3^, with negligible side-effects, as demonstrated by the images of H&E-stained major organs slices	[121]
IR780	DOX	Polydopamine nano clustered micelles (TPGS-IR780@PDA)	Enhanced intracellular accumulation by TPGS (a drug efflux inhibitor)	Nude mice bearing ADR/MCF-7 human breast tumors	Improved tumor-inhibitory efficiency, as evidenced by tumor sizes starting to reduce after 2 days of treatment (8 days for PDT group)	[122]
Ce6	DOX	Polymer–UCNP hybrid micelles (PUHMs)	NIR-triggered	HeLa human cervical carcinoma cells	High cytotoxicity for cancer cells in vitro, due to upconverted emission energy triggering ROS generation and faster DOX release	[123]
Ce6	DOX prodrug (PDOX)	Gd^3+^-loaded copolymeric micelles conjugated with PS	Acid-switchable multimodal imaging (FL, PA, MR) capability	Nude mice bearing ADR/MCF-7 human breast tumors	Notably inhibited the tumor growth and completely eradicated two of the tumors, compared to single therapy; obvious DNA damage and membrane lysis revealed by H&E staining and notable apoptosis of tumor cells revealed by TUNEL staining	[124]
Ce6	DOX	Self-assembled polyethyleneimine–nitroimidazole (PEI–NI) micelles	Hypoxia trigger; PA imaging; tumor targeting by HA	LLC xenograft tumor-bearing mice	Significantly stronger anticancer efficacy than single therapy in vitro, evidenced by IC_50_ value of DOX (1.15 µg/mL) or Ce6 (0.16 µg/mL) in combined group lower than those of chemotherapy (>10 µg/mL) or PDT (0.75 µg/mL); compared therapy showed remarkably prolonged survival after 35 days observation.	[104]
5-(4-Carboxyphenyl)-10,15,20-triphenylporphyrin (Por)	GNA002	Micellar GNA002@cPRP	pH-sensitive; tumor targeting by cRGD; improved drug penetrability in vitro and prolonged tumor-retainability in vivo	HeLa, HN6, A375, MCF-7, and HN30 cancer cells and HeLa tumor-bearing mice	Decreased IC_50_ and increased cell apoptosis for combined group, compared to single therapy, due to increased ROS generation in vitro; tumor weight on day 14 was just 6.3% and 6.7% of that of the saline group of the HeLa and HN6 cancer-bearing mice, respectively, with negligible body weight loss; widespread cancer cell necrosis and apoptosis caused by combined therapy in H&E staining images; highest TUNEL expression and lowest cancer cell proliferation in the TUNEL-staining and Ki-67 staining images, respectively	[125]
Porphyrin	DOX	PEG–PGMA–PDPA Janus macromolecular brushes	Improved drug loading capability by π–π stacking; pH-responsive	4T1 breast cancer mouse model	In vitro studies showed the lowest cell viability (IC_50_: 7.2 µg/mL TPP and 2.5 µg/mL DOX); in vivo studies confirmed that NP-based combination exhibited high phototoxicity and significant tumor inhibition efficacy	[126]
Other Organic Nanoparticles						
Ce6	DTX	Redox-responsive polymer HA–cys-DHA/Ce6 (CHD)	Redox-responsive; Tumor-targeting by HA	MCF-7 breast tumor mouse model	Synergistic antitumor activity in vitro, due to inhibition of microtubule depolymerization, blocking cell cycle, and generating ROS, leading to best antitumor response in vivo	[127]
Ce6	Pt(IV)	Oxygen and Pt(II) self-generating conjugate	Intratumoral self-sufficiency of O_2_	BALB/c mice bearing HeLa, HCT116, and MDA-MB-231 tumors	Enhanced anticancer efficacy both in vitro and in vivo; specifically, in vivo results showed that two of the five mice in combined treatment group were healed, and the tumor volumes of the other three mice decreased to very little	[128]
Ce6	TPZ	Self-assembly PA/HA–Ce6@TPZ NPs	Tumor targeting by HA; dual hypoxia-responsive	Nude mice bearing 4T1 breast cancer	Synergistic anticancer treatment due to PDT-mediated hypoxia-induced cascade TPZ therapy	[129]
Ce6	DOX	DOX-NPs/Ce6-microbubble complex	Local release due to the cavitation of NPs; enhanced extravasation and penetration due to energy of ultrasound	Nude mice bearing MIA-paca-2 human pancreatic carcinoma	Increased therapeutic effects in vitro by cell viability assay and in vivo by normalized tumor volume	[130130]
Ce6	DOX	Hyperbranched polyphosphate SOHNPCe6/DOX	NIR-triggered	Nude mice bearing ADR/MCF-7 human breast tumors	Enhanced in vitro apoptosis inducing efficiency (56.82%) and lower cell viability at 72 h (80.46 ± 6.31%), compared to single-therapy group; high antitumor efficacy in drug-resistant breast cancer nude mouse model	[131]
Ce6	DOX	Ce6/Dox@NPs–cRGD	Tumor targeting by cRDG	MCF-7 xenograft human breast tumors	Significantly shrank tumor volume and prolonged survival time, compared to single therapies, with negligible body weight changes and staining organ slices	[132]
Ce6	DOX precursor (CAD)	Co-assembly LA–CAT–CAD@Ce6 NPs	Tumor targeting by lactobionic acid; pH-sensitive; intratumorally self-sufficiency of O_2_	Nude mice bearing human MCF-7/ADR breast tumor cells	Enhanced cell killing and apoptosis efficiency in vitro and the most effective tumor inhibition and ablation ability	[133]
Ce6	Docetaxel (DTX)	Keratin nanoparticle	Monophasic release	DTX-sensitive HeLa (HeLa-P) and DTX-resistant HeLa (HeLa-R) cells	In monolayers, combined therapy had comparable cytotoxicity to free drugs toward HeLa-P cells, but synergic interaction in HeLa-R cells; induced stronger cytotoxicity and volume reduction rate in spheroids	[134]
Ce6	SN38	Carrier-free nanoparticles (SN38/Ce6 NPs)	Carrier-free	4T1 murine breast cancer cell lines	Significant increase in the inhibition rate by 85%, compared to single therapy, in vitro due to enhanced tumor accumulation and higher cellular internalization	[135]
PheoA	DOX	DOX–PheoA–alginate NPs)	NIR-triggered drug release	B16 tumor-bearing mice	Enhanced tumor growth inhibition by combined therapy with increased serum IFN levels	[136]
PheoA	DOX	Self-assembly PEG–thioketal–DOX NPs	ROS-responsive; phototriggered release	Nude mice bearing CT-26 colorectal cancer	Enhanced anticancer therapeutic effect in vitro by cell viability assay and in vivo by tumor volume change, due to spatiotemporally controlled cascade drug release	[137]
VP	TMZ	Pluronic P85/F127 copolymers	Tumor targeting by biotin	T98-G, U87-MG, and U343 glioblastoma cells	Enhanced antiproliferative effect in vitro via different cell-cycle arrest mechanisms of drug action, especially at low TMZ concentrations and higher light doses	[138]
Hypocrellin B (HB)	PTX	Hyaluronic acid–ceramide nanoparticle	Tumor targeting due to HA	Nude mice bearing A549 human lung adenocarcinoma	Enhanced phototoxicity in vitro and improved anticancer efficacy, by tumor volume change, compared to single PDT and NP-based PDT	[139]
Pyropheophorbide a (PPa)	PTX	Self-assembly heterotypic chemo-photodynamic dimer	ROS-responsive	KB xenograft tumor-bearing nude mice, 4T1 xenograft tumor-bearing BABL/c mice	Synergistic antitumor activity, both in vitro and in vivo	[140]
Carbon dots (CDs)	Metformin (Met)	Traceable DOX/Met/BSA–HA–CDs	Dual-drug system; fluorescence imaging; tumor targeting by HA	MCF-7/ADR human breast cancer cells; S180 murine sarcoma tumor mouse model	Synergistic treatment achieved considerably highest cytotoxicity in vitro and enhanced cancer therapeutic efficiency in vivo, which was attributed to MET reducing the tumor O_2_ consumption, resulting in increased the therapeutic efficiency of oxygen-consumed PDT	[141]
??	DOX	Regenerated silk fibroin-based PC–Mn@Dox-NPs	Multimodality factors responding, resulting in controlled release; intratumoral self-sufficiency of O_2_	4T1 breast cancer mouse model	Enhanced in vitro and in vivo anticancer efficacies, compared to all other combination approaches of PDT and DOX, due to multifactor triggered DOX release and oxygen-dependent PDT enhanced by self-sufficient O_2_	[142]
ICG	Cisplatin (DDP)	Human serum albumin (HSA)–ICG–DDP NPs	NIR-triggered drug release	HSC human oral squamous cell cancer cells and NCM-460 colonic epithelial cells	Improved cytotoxicity for cancer cells in vitro due to higher ROS generation; significantly enhanced tumor growth inhibition compared to 632.06 ± 52.49 mm^3^ in the NP-PDT group and 482.25 ± 42.69 mm^3^ in the NP-chemotherapy group	[143]
ZnPC	DOX	Phthalocyanine-conjugated Glyco-NPs	pH-responsive; good colloidal stability; tumor targeting owing to GLUT5	3T3, MCF7, and MDA-MB-231 human breast cancer cells	High cytotoxicity effect in vitro, due to higher cellular internalization and induction of ROS generation	[144]
ICG	Bromoisophosphoramide mustard intermediate (IPM-Br)	Semiconducting polymer NPs	Light-responsive; intratumoral self-sufficiency of O_2_; NIR imaging	Nude mice bearing 4T1 breast cancer cells	Synergetic anticancer effects due to improved chemo prodrug efficiency (4.3-fold higher, compared with its prodrug-free counterpart) due to PDT-enhanced degree of hypoxia; increased photodynamic efficacy (18-fold higher than ICG)	[145]
Boron-dipyrromethene (BODIPY)	Lenvatinib (VEGFR inhibitor)	Self-assembling NPs (LBPNPs)	pH-sensitive	Human HCC cell lines Hep3B and Huh7	Effectively inhibited tumor growth in vitro by promoting the cascade of caspase apoptotic protease	[146]

#### 4.3.2. Inorganic Nanoparticles-Based PDT Plus Chemotherapy

Inorganic NP-based PDT–chemotherapy combinations have enhanced therapeutic efficiency, due to their high stability, lower degradation rate, and ease of surface modification (Table 3). For example, one group developed a gold-caged organic/inorganic integrating nanoparticle (PTX-PP@Au NPs) encapsulating paclitaxel (PTX). In this multifunctional platform, AuNPs blocked the TRPV6 ion channel in androgen-resistant prostate cancer when under irradiation by NIR (808 nm) laser, and facilitated PTX release obtained an enhanced chemotherapeutic efficiency, both in vitro and in vivo [147].

In addition to gold nanomaterials, up-conversion NPs (UCNPs) have many attractive properties in PDT combined applications, through the conversion of NIR light into UV/Vis wavelengths with high penetration into tumor tissues and lower phototoxicity. Lanthanide ion-doped mesoporous hollow cerium oxide UCNPs loaded with DOX (Ce-UCNPs) were synthesized by Yao et al. for NIR-triggered PDT and chemotherapy treatment of malignant glioma cancer. This nanocarrier is pH-sensitive and intracellular endogenous H_2_O_2_-responsive, resulting in the strong synergistic antitumor efficacy of combined therapy due to accelerated DOX release and self-sufficient O_2_. Remarkable tumor cell viability inhibition has been observed in vitro; 28.2% of tumor cells survived after NP-based combined treatment, compared to 56.1% with DOX-loaded Ce-UCNP without irradiation. In a U87MG malignant glioma cancer mouse model, enhanced tumor growth inhibition and increased apoptosis/necrosis of tumor cells with negligible systemic toxicity were observed [148].

Ceramic NPs are another widely explored delivery vehicle for chemo-PDT; inorganic nanoparticles are often used due to their high biocompatibility and stability. Commonly used inorganic NPs in chemo-PDT include silica (SiO_2_) NPs, titanium oxide (TiO_2_) NPs, and calcium carbonate (CaCO_3_) NPs. DOX-loaded mesoporous TiO_2_ NPs (MTN/DOX) were produced, after which dual targeting components were grafted onto the surface (HA and ADH-1, a cyclic pentapeptide) to synthesize the final formulation This ADH-1-HA-MTN/DOX NP can be photoexcited with UV having a wavelength from 320 to 400 nm. Under X-ray irradiation, TiO_2_ NPs produced ROS to directly kill tumor cells; these effects were further enhanced with higher accumulation of the dual-targeting nanosystem in CD44^high^ tumor cells and EMT process blockage [149]. Furthermore, the shortcomings of X-ray-induced TiO_2_ activation as PDT, including nonspecific harmful effects to normal cells and weak penetration in deep tumor tissues, can be circumvented by loading another PS into the core of the NP. For example, Zhang et al. reported ROS-responsive ZnPC-sensitized TiO_2_ NPs conjugated with chlorambucil (CBL) (mTiO_2_–BCBL@ZnPC NPs). This system, when triggered by NIR, has several advantages, including higher penetration, effective therapeutic effects, biosafety, and low side-effects. Moreover, PDT-generated ROS (H_2_O_2_) will cleave the phenylboronic ester between CBL and the NP, inducing CBL release activation and enabling a spatial and temporal light-triggered combination therapy [150].

SiO_2_ NPs are popularly used for PDT combinations because they are easily surface-functionalized, by adjusting their pore sizes. However, the drawback of silica NPs is that they are sometimes recognized and cleared by the mononuclear phagocyte system (MPS). Thus, several studies have focused on mesoporous SiO_2_ NPs (MSNs) coated with PEG or a membrane layer of erythrocytes, white blood cells, cancer cells, and/or bacteria to improve the application efficiency in cancer therapy [151]. For example, one group generated leukocyte/platelet hybrid membrane-camouflaged dendritic large pore MSNs (LPHM@DDI NPs), loaded with the NIR fluorescent dye IR780 and DOX as a model drug for chemotherapy. The hybrid membrane coating assisted the MSNs to escape from biological clearance, thus extending their circulation time. The tumor-targeting ability was further improved by the LFA-1/ICAM-1 interaction-dependent tumor vascular targeting and crossing effects. As a result, synergistic cytotoxicity and apoptosis-inducing activity were achieved in vitro. Moreover, effective tumor growth suppression and recurrence prevention were achieved in TNBC mice, through the inhibition of cancer cell proliferation and mitigation of angiogenesis [152].

**Table 3:** Preclinical studies on inorganic nanoparticles for codelivery in PDT plus chemotherapy.

PS	Chemo Drugs	Delivery System	Specific Function of Delivery System	Cancer Models	Therapeutic Outcomes of Combination	Ref
Gold NPs						
Au NPs	PTX	PTX-loaded pluronic-PEI@Au NPs	NIR-sensitive; ion channel inhibition	Nude mice bearing PC3 human prostate cancer	Enhanced therapeutic efficiency in vitro and in vivo, with low toxicity on liver function and minimal side-effects to normal organs	[147]
Up-conversion NPs						
CeO_2_ NPs	DOX	Lanthanide ion-doped mesoporous hollow cerium oxide UCNPs (Ce-UCNPs)	pH-sensitive; intratumoral self-sufficiency of O_2_ due to H_2_O_2_-responsive ability	U87MG malignant glioma tumor mouse model	Remarkable cell viability inhibition in vitro and tumor growth inhibition, compared to treatment with DOX or PDT, with negligible systemic toxicity (little body weight difference between groups)	[148]
ZnFe_2_O_4_	Pt(IV) prodrugs	UCNPs–Pt(IV)–ZnFe_2_O_4_, denoted as UCPZ	Multimodality bioimaging (UCL, CT, MRI, and PA); inhibited biological clearance; enhanced tumor accumulation	U14 cervical tumor mouse model	Significantly enhanced antitumor effect in vivo	[153]
ZnFe_2_O_4_	DOX	UCNPs with a mesoporous ZnFe_2_O_4_ shell (UCNPs@mSiO_2_)	Trimodal imaging (CT, UCL, MRI)	HeLa xenograft cervical tumor mouse model	High anticancer effectiveness both in vitro and in vivo	[154]
Rose Bengal (RB)	DOX	UCN@mSiO_2_-(Azo + RB) nanoimpellers	Faster drug release due to Azo molecules	HeLa human cervical carcinoma cells	High cytotoxicity effect for cancer cells in vitro	[155]
Rose Bengal (RB)	Pt(IV) prodrugs	Biocompatible core–shell–shell UCNPs (PEG/RB-Pt(IV)-UCNPs)	NIR-triggered drug release	A2780 and A2780cisR human ovarian cancer cells	Improved cytotoxicity for both cisplatin-sensitive and -resistant human ovarian cancer cells in vitro	[156]
Rose Bengal (RB)	DOX	Cancer cell membrane (CM)-cloaked UCNPs	ROS-sensitive; inhibited biological clearance; enhanced tumor accumulation	Primary 4T1 murine model; Metastatic Luc-4T1 breast orthotropic tumor model	Enhanced uptake in tumor cells and deeper penetration in spheroids; strong synergistic antitumor efficacy and synchronously causes increased DAMPs release, leading to tumor-specific immunity; when combined with anti-CD73 antibodies, had a better effect on lengthening the period of survival and inhibiting lung metastasis than monotherapies associated with stronger systemic cytotoxic T-cell responses	[157]
Rose Bengal (RB)	DOX	NIR-triggered ROS-sensitive (UCN/SiO_2_-RB + DOX) @PPADT NPs	NIR-triggered drug release	HeLa human cervical carcinoma cells	Achieved a better inhibitory effect on cancer cell in vitro at concentrations over 100 mg/L than single therapy	[158]
RBHA	Pt	CaF2: Yb^3+^/Er^3+^ UCNPs coated with NaGdF4 shells (UCNPs–RBHA–Pt–PEG)	Multimodality bioimaging (UCL, MRI)	CT26 murine colorectal carcinoma cells	Visibly decreased tumor sizes for combined therapy group at a low irradiation power density (0.35 W/cm^2^, 6 min)	[159]
Methylene blue (MB)	DOX	NaYF4:Yb,Er UCNPs	Tumor targeting due to anti-HER2 peptide	SKBR-3 (HER2-positive) and MCF-7 (HER2-negative) breast cancer cells	Significant decline in the cell viability by 95%, compared to 77% for chemo-drug and 84% for PDT only in vitro; cell viability was suppressed by 66% in a 3D model of SKBR-3 tumor spheroids, due to improved uptake of NPs	[160]
ZnPc	DOX	Protein–polymer bioconjugate-coated multifunctional UCNPs	Excellent water solubility, good stability, and low toxicity; real-time imaging capability	HeLa human cervical carcinoma cells	Enhanced tumor cell killing efficiency in vitro	[161]
Ce6/ZnPc /methylene blue (MB)	DOX	Red-emitting up-converting nanoparticles (α-CD-UCNPs)	-	A549 human epithelial lung cancer cells	Higher therapeutic efficacy, relative to the individual means, for cancer therapy in vitro	[162]
Polyelectrolyte brushes (PFNS)	AQ4N	pH-sensitive Mn-Ca_3_(PO_4_)_2_ (MnCaP) layer-coated UCNP@PFN	pH-sensitive; hypoxia-activated; multi-imaging (MRI, FL, UCL)	HeLa human cervical carcinoma cells	Enhanced therapeutic effect, thereby reaching a tumor inhibition rate as high as 83%; highest level of cell apoptosis, as evidenced by H&E staining of tumor slices	[163]
Graphene oxide (GO)	DOX	UCNPs–DPA–NGO–PEI–DOX	UCL imaging; improved drug loading capability	U14 murine liver cancer xenograft tumor mouse model	Substantially superior cell killing effects in vitro, due to sensitive disulfide bond; higher tumor inhibition efficiency than monotherapies	[164]
UCNPs	DOX	Core/shell structure SPTP@UCNP-RB NPs	NIR-controlled; tumor targeting to E-selectin; intratumoral self-sufficiency of O_2_	Multicellular spheroid model; 4T1 murine breast cancer model	Synergistic anticancer effects and improved ICD levels in cells; enhanced uptake, penetration, and antitumor efficacy against multicellular spheroids; synergistically destroyed the orthotopic tumors and efficiently suppressed lung metastasis by cascade-amplifying systemic antitumor immunity through induction of ICD with CD8^+^/CD4^+^ T-cell infiltration and IL-6/IL-10 secretion	[165]
Ceramic Nanoparticles (Silicon dioxide Nanoparticles)						
Ce6	Pt(IV) prodrugs	MSNs/Ce6/Pt	Biocompatibility and stability; higher cellular uptake	Cisplatin-resistant A549R lung cancer cells	Improved treatment efficiency due to elevated cellular ROS level in vitro	[166]
Ce6	DOX	Erythrocyte-mimetic MSNs (RMSNs-Dox/Ce6)	Biocompatibility and stability; high loading capacities; irradiation sensitive; inhibited biological clearance; enhanced tumor accumulation	4T1 breast tumor mouse model	Effective cell killing ability, up to 92.1% cell death after treatment, compared to 75.2% in the NP-based chemotherapy group; enhanced tumor inhibition rate (91.4%), which was significantly higher than PDT single (68.9%) and chemotherapy single (73.7%) therapy, respectively; inhibited 75.1% metastatic foci to lung, which was more effective than monotherapies	[167]
TMPyP	DOX	MSN@SiNPs@TMPyP-FA	Biocompatibility and stability; biological autofluorescence; tumor targeting by HA	MCF-7 human breast carcinoma cells and A549 human lung cancer cells	High cytotoxicity for tumor cells in vitro	[168]
IR780	DOX	Leukocyte/platelet hybrid membrane-camouflaged dendritic large pore MSNs (LPHM@DDI NPs)	Biocompatibility and stability; tumor targeting by P-selectin/CD44 binding; inhibited biological clearance; enhanced tumor accumulation	4T1 breast tumor mouse model	Synergistic cytotoxicity and apoptosis-inducing activity in vitro; effective tumor suppression and recurrence prevention in vivo through directly killing tumor cells and indirect anti-angiogenesis	[152]
ICG	TPZ	Erythrocyte and tumor cell membrane camouflaged MSNs (IT@MSN@RTM)	Biocompatibility and stability; inhibited biological clearance; enhanced tumor accumulation; irradiation sensitive	4T1 breast tumor mouse model	1.3 times tumor inhibition rate of combined therapy, compared to 47% in the PDT treatment group alone	[169]
HCE6	OXP	OH-MSNs	Biocompatibility and stability; pH-sensitive	Nude mice bearing FRH0201 human hilar cholangiocarcinoma	Enhanced proliferation-inhibitory effects and killing effect of oxaliplatin in NPs in vitro; much more effective in inhibiting tumor growth in vivo compared with O-MSNs	[170]
Tellurium (Te)	PTX	Double hydroxide gated MSNs (MT@L-PTX@FA)	Biocompatibility and stability; sustained release; pH-sensitive; tumor targeting by FA	HepG2 human hepatocyte carcinoma cells	Enhanced cancer cell killing effects in vitro by increased ROS generation	[171]
IR820	TPZ	Glutathione decomposable MSNs (GMONs)	Biocompatibility and stability; GSH/enzyme dual-responsive; tumor targeting by HA	4T1 breast tumor mouse model	Enhanced tumor inhibition rate of dual-loaded nanohybrids was up to 76% under NIR laser irradiation in vivo, due to PDT-induced hypoxia resulting in improved TPZ effects	[172]
Hematoporphyrin (HP)	DOX	CeO_2_ NPs coated dual-loaded MSNs (MSN-HP-DOX@CeO_2_)	Triple-sensitive (GSH, pH, and light irradiation)	HeLa human cervical carcinoma cells	High cytotoxicity to cancer cells, due to the more controllable DOX release under triple factors	[173]
Si-Pc	DOX	^68^Ga-labeled magnetic-NIR persistent luminescent hybrid MNPs (DOX/Si-Pc-loaded HMNPs)	Trimodal imaging (NIR-PL, PET, MRI)	Nude mice bearing LNCaP human prostate cancer cells	Outstanding cancer cell killing ability in vitro and tumor suppression effect in vivo, due to prolonged NPs retention and DOX release in tumor area	[174]
Ceramic Nanoparticles (Titanium Oxide Nanoparticles)						
Au@TiO_2_ NPs	DOX	Zwitterionic polymer-gated Au@TiO_2_ core-shell nanoparticles	NIR-sensitive; MRI imaging; improved hemocompatibility of NPs; prolonged circulation time.	Nude mice bearing HeLa human cervical carcinoma	Both in vitro and in vivo anticancer experiments demonstrated that the tumor was effectively inhibited, with few side-effects	[175]
ZnPc	Chlorambucil (CBL)	TiO2 nanoparticles (mTiO_2_-BCBL@ZnPC NPs)	NIR-triggered; ROS-triggered; intratumoral self-sufficiency of O_2_	MCF-7 human breast cancer cells	High cytotoxicity effect for cancer cells in vitro due to higher cellular internalization and induction of ROS generation	[150]
TiO_2_	DOX	Mesoporous TiO_2_ ADH-1–HA–MTN/DOX NPs	Tumor dual targeting by CD44 and N-cadherin; irradiation by X-ray	A549 human non-small-cell lung carcinoma cell line	Enhanced cancer cell killing effects and cell inhibition rate in vitro by increased ROS generation; potential to overcome drug resistance problem by preventing EMT process	[149]
Magnetic Nanoparticles						
Si-Pc	DOX	^68^Ga-labeled magnetic-NIR persistent luminescent hybrid MNPs (DOX/Si-Pc-loaded HMNPs)	Trimodal imaging (NIR-PL, PET, MRI)	Nude mice bearing LNCaP human prostate cancer cells	Studies with mice tumor models demonstrated that the NP-based combination possessed excellent cancer cell killing ability and an outstanding tumor suppression effect without systemic toxicity, which is associated with prolonged tumor retention of NPs and the durable release of loaded DOX within tumor tissues	[174]
CuS NPs	DOX	Hollow mesoporous CuS NPs capped with magnetic iron oxide NPs (HMCuS/DOX@IONP-PEG)	Controlled drug release; magnetic targeting; property and MR imaging	Nude mice bearing MCF-7 human breast cancer cells	Improved treatment efficiency due to increased drug levels at tumor site and elevated cellular ROS level in vivo; reduced cardiotoxicity of DOX in NPs than free drug	[176]
ICG	Pt(IV) prodrugs	MoS_2_ nanoflowers (MoS_2_@Fe_3_O_4_-ICG/Pt(IV))	Trimodal imaging (MR, IR, PA)	L929 fibroblast cells or Hela cells, H22 live cancer mouse model	Enhanced antitumor efficacy by both in vitro and in vivo assays	[177]
Ce6	Celastrol (CSL)	Manganese/iron-based nanoprobes (Fe_3_O_4_@MnO_2_-CSL/Ce6)	pH-responsive; intratumoral self-sufficiency of O_2_; T1/T2 MRI and PA imaging	Nude mice bearing Bel-7402 human hepatocellular carcinoma cells	Synergistic therapeutic effects for tumor inhibition through improving the tumor hypoxic environment, thereby enhancing PDT effects	[178]
ICG	DOX	MnO_2_-coated silk fibroin NPs (SF@MnO_2_/ICG/DOX)	Intratumoral self-sufficiency of O_2_; dual imaging (FL and MRI)	4T1 breast tumor mouse model	Significant tumor inhibitive efficacy, with a tumor growth inhibition rate of 89.6%, compared to moderate tumor inhibition effect of single therapies at 14 days; H&E staining, TUNEL assays, Ki67, DHE, and HIF-α IF staining of the excised tumor sections were subsequently performed, in order to evaluate the tumor tissue destruction	[179]
Calcium Carbonate Nanoparticles						
ICG	TPZ	Hybrid CaCO_3_/TPGS nanoparticles	Tumor targeting by RGD peptide	Subcutaneous U87MG and orthotopic B16F10 tumor-bearing mouse model	Intensive effects in vitro and in tumor inhibition, with negligible side-effects	[180]
Metal-Organic Framework-Based PDT plus Chemotherapy						
Porphyrin	DOX	ZnO-gated porphyrinic MOF-AS1411	pH-sensitive; Tumor targeting by nucleolin-specific AS1411 aptamer	Nude mice bearing human HeLa human cervical carcinoma cells	Highly efficient cancer cell killing and tumor inhibition; tumor ablation was also even achieved, without undesirable side-effects	[181]
RuII polypyridyl alkyne complex (Ra)	DOX	UiO–Ra–DOX–CuS	pH-sensitive; NIR-triggered drug release; intratumoral self-sufficiency of O_2_	MDA-MB-231 human breast cancer cells	Improved cytotoxicity for cancer cells in vitro than chemotherapy alone (69% vs. 42%)	[182]
Photochlor (HPPH)	AQ4N	Azido-/PS-terminated UiO-66-H/N3 NMOFs	Hypoxia-triggered; enhanced dispersion by PEG layer	Nude mice bearing U87MG human glioblastoma cancer	Enhanced therapeutic efficacy with negligible systemic toxicity due to PDT and hypoxia-activated cytotoxicity of AQ4N	[183]
Ce6	Gambogic acid (GA)	MnO_2_-based core–shell GC@MCS NPs	Hypoxia-triggered; intratumoral self-sufficiency of O_2_; increased penetration; tumor-targeting by HA	4T1 mammary tumor models	Superior potency and efficacy in tumor regression; 92.41% of 4T1 tumor inhibition rate	[184]
Au@TiO_2_ NPs	DOX	Polymer-gated Au@TiO_2_ core–shell nanoparticles	NIR-sensitive; MRI imaging; improved hemocompatibility of NPs; prolonged circulation time	Nude mice bearing HeLa human cervical carcinoma	Both in vitro and in vivo anticancer experiments demonstrated the tumor was effectively inhibited, with minimal side-effects, by the multifunctional NPs	[175]
ICG	TPZ	Zeolitic imidazolate framework-8 (ZIF-8) coated ZnS NPs (ZSZIT)	Hypoxia-activated; H_2_S-sensitive cascade	Nude mice bearing Huh7 human hepatoma	Synergistic antitumor effect both in vitro (by CCK8 assay) and in vivo (by tumor volume change)	[185]
Other Inorganic Nanoparticles						
octaethylporphine (OEP)	Cis-(PEt_3_)_2_Pt (OTf)_2_ (cPt)	Metallacage-loaded NPs	Tumor targeting by cRGDfK; enhanced tumor accumulation and cellular internalization ability	Nude mice bearing A2780/A2780CIS ovarian tumor	Highest antitumor outcome, with 89.2% tumor inhibition rate, compared to 14.1%, 25.5%, and 66.8% for chemo, NP-chemo, and NP-PDT, respectively; decreased the hepatotoxicity and nephrotoxicity of the platinum-based anticancer drug	[186]
TPP	Cis-(PEt_3_)_2_Pt (OTf)_2_ (cPt)	Metallacage-loaded NPs	Enhanced penetration into drug-resistant 3D tumor spheroids	HuH7 human hepatocellular carcinoma cells and CCLP-1 intrahepatic cancer cells	Enhanced ability to decrease tumor cell mobility and sphenoid formation; CSCs from these spheroids have a lower tumorigenicity, compared to CSCs in the spheroids after single therapy	[187]
ICG	DOX	Hollow mesoporous Prussian blue (HMPB)@PEI/ICG/DOX)	FL imaging due to ICG	4T1 tumor-bearing mouse models	Effective tumor inhibition effect with a tumor growth inhibition rate of 95.5%, while single therapies did not effectively suppress tumor growth in the long term; insignificant short-term toxicity or damage to normal tissues	[188]
NPs	DOX	Hollow CuS nanocubes (CuS@PEG)	NIR-triggered; pH-sensitive	HepG2 human hepatocyte carcinoma cells	Enhanced specific cytotoxicity to cancer cells in vitro	[189]
NPs	DOX	Silver NPs	pH-sensitive; intracellularly probed; tumor targeting by FA	SKOV-3 and L1210 cells	Enhanced toxicity in vitro	[190]

### 4.4. Nanoparticle-Based PDT Plus Immunotherapy

**Figure 6:**
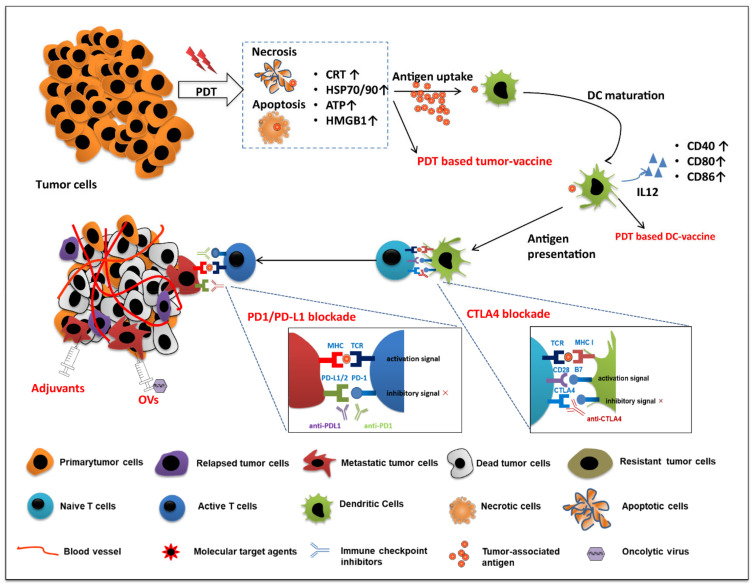
NP-mediated photodynamic therapy in combination with various immune therapies, including vaccination, immune checkpoint inhibitors, oncolytic viruses (OVs), and adjuvants. The combination works to enhance the key factors of the immune-oncology cycle—antigen release, antigen presentation, APC activation, T-cell activation, T-cell infiltration, and antigen recognition—to kill primary tumor cells and overcome tumor recurrence and metastases.

Cancer immunotherapy has been widely explored, both alone and in combination with other therapies. The US Food and Drug Administration (FDA) has approved it for nearly 20 different types of cancer treatments, due to its durable and robust effects. Immunotherapy can be classified into five distinct strategies: nonspecific immune stimulation (cytokines, Toll-like receptors (TLRs) ligands), vaccination, adoptive cell transfer, checkpoint blockade, and tumor antigen–antibody targeting (Figure 6). A nano technique-based combination of PDT and immunotherapy can improve the therapeutic ratio, prevent drug leakage, and minimize the shortcomings of a single modality [191].

Integrating PDT with immunotherapy in nanoparticles (Table 4) enables the eradication of both the primary tumor and the metastatic cancer cell growth. During treatment, PDT first effectively clears the primary tumor(s) by inducing immunogenic cell death. Subsequently, PDT-induced dying tumor cells are regarded as new tumor-derived antigens, which can be phagocytosed by macrophages and dendritic cells. In addition, increased stress protein expression and DAMP release from tumor cells lead to acute inflammation and leukocyte infiltration, as well as maturation activation of dendritic cells. However, some studies have shown that these series of immune responses are insufficient to inhibit escaped tumor cells. Myeloid-derived suppressor cells (MDSCs) infiltrate the tumor, release anti-inflammatory cytokines, and activate regulatory T cells (Tregs) to inhibit the antitumor immune response. Thus, the escaped tumor cells can survive and recover again [192]. In addition to PDT-induced antitumor immunity, nanoparticle-based combined photo-immunotherapy can modulate the immune system against survival/metastatic tumor cells, by decreasing immunoregulatory suppression (immune checkpoint blockade therapy) or increasing immunogenicity of the tumor microenvironment (utilizing immunoadjuvants), eventually attracting more antigen-presenting dendritic cells [71].

#### 4.4.1. Nanoparticle-Based PDT Plus Immune Checkpoint Blockade Therapy

Nanotechnology-supported PDT combined with immune checkpoint therapy, including anti-PD-1/PD-L1 and anti-CTLA-4, has been reported to have synergistic antitumor effects [218]. Nanoparticles have been proposed to decrease the required therapeutic doses, minimize the risk of serious systemic toxicity, and prolong the immune response [219]; for example, it has been demonstrated that loading anti-CTLA-4 monoclonal antibodies in hydrogel micelles [220] or PEGylated liposomes [221] decreased the associated toxicity in healthy organs.

Additionally, PS-based nanoparticles plus immune checkpoint inhibitors are capable of switching off the inhibitory antitumor pathways between T cells and tumor cells and normalizing the suppressive tumor microenvironment state. NPs help to enhance the PDT-induced immunomodulation at the same time with PDT, thereby facilitating complete tumor ablation and inhibiting tumor relapses and metastasis. Zheng et al. generated a novel Janus nanoparticle that combined PDT and magnetic hyperthermia, a type of thermal cancer treatment using magnetic nanoparticles to generate heat [222] while performing a CTLA4 blockade. These nanoparticles improved the levels of ICD and significantly decreased primary tumor weights and the number of pulmonary metastatic nodules in MCF-7 tumor-bearing mice [200].

NP-based PDT combination with commonly used immune checkpoint inhibitors, including anti-PD1 or anti-PD-L1 antibody, shows the potential to inhibit the dissemination of tumor, its relapse, and metastasis by enhancing systemic antitumor immune responses. Yan et al. utilized polydopamine-encapsulated UCNPs with surface-loaded PS (Ce6), which significantly enhanced the antitumor efficacy of PDT in the primary tumor and prolonged survival time of a 4T1 tumor-bearing mouse model. In addition, combined PDT and PTT therapy using these NPs enhanced ICD levels and systemic antitumor immune responses. The effect was further improved by the combination with anti-PD1 antibody, through the enhanced activity of macrophages, B cells, CD4^+^/CD8^+^ T cells, IFN-γ-expressing CTLs, and effector memory T-cells [198]. Another study, which used both 4T1 and TUBO (a cloned BALB-neuT mouse mammary carcinoma cell line) bilateral syngeneic mouse models, showed that combined NP-mediated PDT with PD-L1 antibody completely eradicated the tumor, and there was a low (0.4%) metastasis rate of tumor nodules in the lungs [214].

#### 4.4.2. Nanoparticle-Based PDT Plus Vaccination and Immunoadjuvants

**Figure 7:**
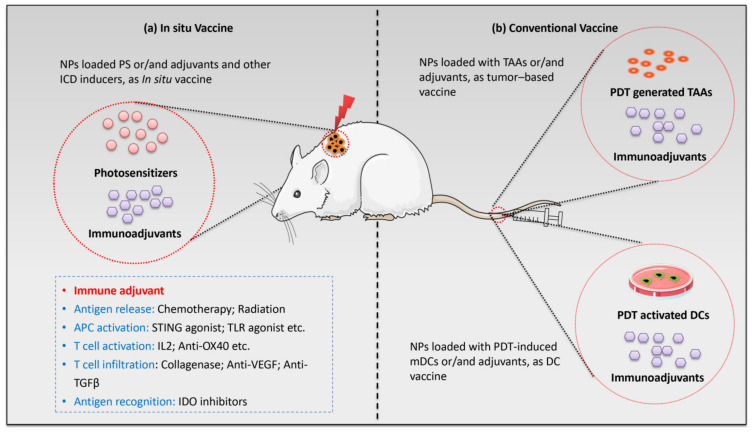
Nanoparticle-based PDT generated vaccines: (**a**) NPs carrying PS and/or immune adjuvants accumulate in tumor tissue after administration. Through irradiation, PDT itself will supply tumor antigens; (**b**) NPs carrying PDT-generated tumor antigens or ex vivo stimulated mature DCs (and/or together with immune adjuvant) are administered systemically as conventional vaccines. This activates and expands effector T cells for vaccine antigen-specific systemic responses. With the help of immune adjuvants and other ICD inducers, strong systemic antitumor immunity against all tumor antigens will be induced, and PDT treatment will ablate surviving tumor cells and metastatic cells.

Therapeutic cancer vaccines are based on targeting specific tumor-associated antigens (TAA) and adjuvants, in order to enhance the cancer antigen presentation of antigen-presenting cells (APCs) and the activation of T cells [223]. As shown in Figure 7, in the context of PDT–immuno-vaccine therapy, studies have focused on the systemic delivery of PDT-treated tumor cell debris as antigens and/or immunoadjuvants, defined as conventional vaccines. Moreover, accumulated NPs loaded with PS and adjuvants have been reported to induce a strong ICD by providing irradiation as an in situ cancer vaccine [224].

##### Nanoparticle-Based PDT-Generated Conventional Vaccines

Regarding PDT-generated tumor cell vaccines, tumor cells are treated by PDT ex vivo to induce necrosis and apoptosis. This leads to an increased expression of heat-shock proteins on the tumor cell surface and the generation of neo antigen specific antigens [225]. Thus, they may have the ability to fight the same tumor or tumors with inherent low immunogenicity [6,226]. The anticancer efficiency of vaccines can be further enhanced by appropriate drug delivery systems and adjuvants. NPs protect antigens from rapid degradation or elimination before they achieve their robust and long-term therapeutic effects. For example, a hydrogel-delivered PDT–tumor cell vaccine successfully delayed tumor growth kinetics and prevented the relapse of tumors. Weak bioluminescence signals in lungs and apoptosis/necrosis in collapsed tumor tissues by H&E staining were detected in the hydrogel-based vaccine group. These results demonstrated that the hydrogel vaccine cooperating with PDT induced stronger antitumor immunity than PDT alone [196]. Interestingly, it has been reported that the quality of a PDT-based tumor cell vaccine is also affected by other factors; for example, regulatory macrophages (Mregs, anti-inflammatory macrophages subset) impeded the antitumor immune response activation by a temoporfin-PDT-/Ce6-PDT-mediated vaccine—these kinds of vaccines are generated using PDT-treated tumor cells as antigens to stimulate the immune system to kill the cancer cells. However, it has been shown that the inhibitory anticancer effect of Mregs can be relieved through the use of antibodies with Mreg immunodepleting properties (e.g., anti GR1 antibody) in squamous cell carcinoma SCCVII tumor models [227,228]. On the basis of such explorations, it is possible to design and prepare nanoparticles combined with inhibitors or antibodies, in order to generate better PDT-based tumor cell vaccines in the future.

In addition to PDT-generated tumor cell vaccines, investigators developed a PDT-induced therapeutic dendritic cell (DC) vaccination by tumor-specifically triggering DC activation and IL12 expression [229]. They immunized SKH-1 mice with DCs after stimulation by PDT-treated PECA cells. A complete inhibition of tumors was observed in the PDT-DC vaccine group at 21 days after rechallenging vaccinated mice with the same PECA tumor cells.

The combination of PDT with other conventional vaccines (e.g., peptide and genetic vaccines), such as TLR5 agonist flagellin-adjuvanted tumor-specific peptide vaccination (FlaB-Vax) has revealed that this combination can enhance the infiltration of antigen-specific CD8^+^ T cells, effector memory CD8^+^ T cells, and IFN-γ expression in a B16-F10 tumor-bearing model [230]. Furthermore, combination treatment of PDT with a synthetic long peptide (SLP) vaccine, covering T-cell (CD4 and CD8) epitopes of tumor antigens (i.e., from the human papillomavirus (HPV)16 E7 oncoprotein), has been regarded as a prospective treatment method for oncogenic virus-induced cancers (e.g., HPV or leukemia virus). This has been proven in murine TC-1 and RMA tumor models by enhanced local tumor ablation and robust systemic immune responses after combination therapy of SLP vaccine and PDT [231].

##### Nanoparticle-Based PDT-Induced In Situ Vaccines

In addition to these conventional vaccination strategies, PDT has a vaccine effect in situ, due to the induced ICD. In peritoneal mesothelioma, a study has shown that a PDT-induced in situ DC vaccine led to highly significant survival in vivo. Moreover, the antitumor immune responses of the vaccine were enhanced when combined with CTLA4 blockade. In particular, this combined treatment regimen stimulated the proliferation, cytotoxic effects, and activation of CD4^+^/CD8^+^ T cells, with a more rapid migration toward the lymph nodes than a traditional LPS-induced DC vaccine [232]. Furthermore, a combination of ICD-inducing therapies (chemotherapy and radiotherapy) with nanoparticles greatly improved the immunogenicity and downstream immune responses of the PDT-induced immune vaccine. For example, a nanoscale chimeric crosslinked polymersome (CCPS) composed of PS and DOX for TAA secretion to produce in situ DC vaccination showed better MC38 tumor growth inhibition and lower distant tumor formation due to DC activation enhancement by CCPS and PDT [206]. Another innovative discovery was the combined intervention of an oncolytic viral vaccine (in situ vaccine) and protoporphyrin IX (PpIX)-mediated PDT. This resulted in complete cell death in a human pancreatic cancer cell line (PsPC-1 and BXPC-3), when compared to 30% single PDT-induced cell death [233].

Additionally, NPs themselves can intrinsically stimulate immune responses as adjuvants, such as positively charged polymers containing primary, secondary, or tertiary amines. Yang et al. reported a chimeric crosslinked polymersome acting as an adjuvant by inducing proinflammation factor release and activating the stimulator of interferon genes (STING)-dependent pathway. The system simultaneously combined PDT, DC vaccine, and chemotherapy (DOX). It enhanced the tumor abscopal effect in primary and distant colon MC38 tumors by increasing DC maturation in lymph nodes and CD8^+^ T cells in the tumor(s) [206]. Taken together, PDT combined vaccines are an effective way to induce host immunity against the primary tumor and tumor relapse; as such, they are currently being assessed in both preclinical and clinical trials.

##### Enhanced Vaccination Effects by Immunoadjuvants in Nanoparticles

Similar to antigens, the limitations of adjuvants include rapid degradation, clearance, and ineffective cellular uptake. Nanoparticles supporting vaccines can load PS and immunoadjuvants synchronously, in order to minimize systemic side-effects and to enhance the antitumor efficacy of the two therapies [234]. The immunopotentiators in the nanosystems may enhance the PDT-induced immune responses by targeting APCs and acting as in situ vaccines to modulate tumor growth. There are various adjuvants in cancer immunotherapy, defined as immunotherapeutic agents targeting tumor cells, T cells, and ligands for pattern recognition receptors (PRR), which have been reported to induce immune responses [235].

Some commonly used APC-targeting immunoadjuvants in clinical practice have been successfully tested to act as partners to PDT. Imiquimod (R837) has the capability to activate DCs and B cells to induce cytokines for Th1 cell immunity and facilitate antibody production [236]. Xu et al. synthesized multitasking UCNP–Ce6–R837 nanoparticles. This nanoparticle triggered robust immune responses, including enhanced DC activation, enriched effective T-cell population, and long-term immune memory, to inhibit primary and distant tumor growth and to prevent tumor recurrence [199]. Notably, PDT treatment of large established tumors enhances the uptake of NPs in the tumor, which accumulates in the myeloid cells in the tumor microenvironment [29]. Moreover, PDT combined with intratumoral injection of immunostimulatory NPs encapsulated with TLR3-/TLR7-ligands and chemotactic agent MIP3alpha synergizes in local and distant antitumor effects [237]. In addition, a chitosan-derived immunoadjuvant has been shown to synergistically enhance the immune response during PDT irradiation [238]. CpG oligodeoxynucleotides (ODNs) contain unmethylated CpG motifs, which can boost immune responses (e.g., DC activation and local inflammation) after PDT, by triggering cells that express Toll-like receptor 9, including dendritic cells and B cells [239]. Cai et al. reported metal–organic framework (MOF)-based nanoparticles coloaded with PS and CpG adjuvant. This nanoparticle strongly enhanced the level of TAAs and led to in situ DC vaccination, in order to inhibit primary tumor growth, HIF-1α-induced survival, and metastasis in a H22 mouse model [208]. Zhang et al. utilized pH-responsive metallic core–shell composite nanoparticles consisting of copper sulfide coated with a mesoporous silica (mSiO_2_) shell, CpG ODNs, and PS (PpIX). In addition, the NP-based combination showed remarkable anticancer effects by overcoming the limitation of the hypoxia tumor environment due to PDT antitumor efficacy (MnO_2_ can decompose PDT-generated H_2_O_2_ into oxygen) and enhanced CTL infiltration and IFN-γ production by CpG ODNs in the tumor. Furthermore, their results showed that this NP, when combined with PD-L1 blockade therapy, has the potential to inhibit metastasis of tumors [212]. Several other APC-targeting immunoadjuvants may work as promising therapeutic supporters for PDT, such as TLR2 agonists (CL401/CL413/CL429), an activator of the proinflammatory transcription factor NF-κB (Pam3CSK4), and agonists of the stimulator of IFN genes (STING). Significant efforts are required to explore the combination with PDT for personalized therapy design.

Moreover, the combination of PDT and T-cell activators which intensify the direct activation of T cells (e.g., anti-OX40, IL-2, and anti-CD3/28) or therapeutic cargoes affecting T-cell infiltration into tumor tissues (e.g., collagenase, anti-VEGF, and anti-transforming growth factor (TGF-β) is also a promising strategy to improve the treatment efficiency. David et al. showed that T cells activated by anti-CD3 and anti-CD28 antibodies display an increased sensitivity to Pc4-PDT-induced apoptosis (10.6–81.2%), indicating the potential of combining PDT with T-cell agonists [240]. Ling et al. constructed hollow mesoporous organosilica nanoparticles (HMONs) encapsulated with collagenase (Col); they could degrade the collagen I fiber in the extracellular matrix (ECM) to normalize the tumor immune suppression environment, before being hybridized with the PS HPPH. Nanoparticle HMONs have been employed as delivery systems with excellent loading capacity, biocompatibility, and biodegradability. They were shown to have better antitumor effects in a tumor mouse model than PDT alone. Immunofluorescence characterization of tumor tissues demonstrated the degradation of ECM after treatment, which was linked to increased immune response and O_2_ infiltration into tumor tissues [241]. More agents that target T-cell infiltration combined with PDT were discussed in a previous section (targeted therapy). Taken together, studies that focus on PDT combined with immunologic adjuvants are currently limited, and it will be worthwhile to further explore these as novel treatment options.

#### 4.4.3. PDT Plus Nonspecific Immune Stimulation

Cytokines have proven to be a novel therapeutic approach in treating patients with advanced malignancies. Interferon-γ (IFN-γ), interferon-α (IFN-α), interleukin-2 (IL2), tumor necrosis factor-α (TNFα), and interleukin-12 (IL12) are the most successful therapeutics approved for clinical use. Some of these cytokines have shown enhanced antitumor effects when combined with PDT therapy; for instance, PDT in combination with vitamin D3-binding protein-derived macrophage-activating factor (DBPMAF) showed enhanced tumor-inhibitory effects by inhibiting angiogenesis [242]. Moreover, the tumor-controlling effects of PDT were potentiated by the intraperitoneal administration of recombinant human TNF-α in mice [243]. Administration of granulocyte-macrophage colony-stimulating factor (GM-CSF) functioned by increasing macrophage infiltration to enhance PDT-induced effects [244]. However, the relatively high toxicity of cytokine therapy and the complex tumor environment limit its usage in clinical settings. Hence, through the integration of PDT and cytokines in nano-frames, the essence of the two therapies can be achieved, and the shortcomings of cytokine therapy can be mitigated. Cytokines in NPs can generate an optimal immune response to help PDT eradicate both the solid and the metastatic tumor. However, few studies have been performed, and more investigation is warranted to explore how delivery systems assist in combined PDT–cytokine therapy.

### 4.5. Nanoparticle-Based PDT in Cancer Theragnostic

From metal complexes to polymeric nanoparticles, NP-based PDT combination platforms have been employed for multimodal imaging (e.g., magnetic resonance (MRI), photoacoustic (PA), positron emission tomography (PET), and computed tomography (CT)) and diagnosis systems, due to the inherent fluorescence of PSs, the physical properties of NPs, and the use of doped contrast agents (i.e., a medium that can increase the contrast of internal body structures or fluids in medical imaging).

In particular, formulations that are based on AuNPs, superparamagnetic NPs (SPIONs), graphene oxide (GO), and carbon nanodots (CDs), among others, have the potential to combine diagnosis, monitoring, and therapy in the same nanoplatforms [245]. As discussed above, ^64^Cu-labeled lipoprotein-mimicking NPs could provide preoperative PET/CT imaging for primary tumor localization and intraoperative fluorescence imaging for the visualization of tumors and the subsequent lymphatic drainage network status 24 h after intravenous injection in a VX-2 buccal carcinoma rabbit model [82]. In addition to labeling with ^64^Cu, ^18^F, ^124/125^I, and ^125^Cd can act as PET contrast agents for nanoparticles. Moreover, researchers have developed hyaluronic acid-modified Au nanocages (AuNCs-HA), allowing for thermoelastic expansion-induced imaging upon PA waves, thus integrating imaging and RT-PDT therapy into one platform [88]. This may lead to future studies focused on the combination of imaging-guided surgery/RT with PDT.

NPs based on the use of paramagnetic ions (e.g., Gd^3+^, Mn^2+^, Dy^3+^, Fe^3+^, and Ho^3+^) have the ability to facilitate MRI imaging [246]; for example, core–shell structured NaGdF_4_:Yb-UCNPs containing Pt(IV) prodrugs were modified with polyethyleneimine (PEI), conjugated with DSP molecules and ZnFe_2_O_4_–dopamine (ZnFe_2_O_4_–DA) NPs, and subsequently coated with a PEG layer. Therefore, this platform integrates UCL, CT, MRI, and PA, thus serving as a multimodal bioimaging system [153]. Hybrid metal NPs based on mesoporous silica NPs, integrating NIR-PLNPs (Ga_2_O_3_:Cr^3+^, Nd^3+^), magnetic nanoparticles (Gd_2_O_3_), and radionuclides (^68^Ga) in one constructor, were named DOX/Si-Pc-loaded HMNPs. This all-in-one system, developed by Rui et al., possesses the advantages of long-term trimodal imaging ability (NIR-PL, PET, and MRI) with the synergistic tumor inhibition effect of chemotherapy and PDT [174], proving an ideal nanoplatform for combination cancer theragnostic and cancer therapy.

## 5. Concluding Remarks and Future Outlook

In conclusion, the increasing incidence of patients with advanced cancer, postoperative recurrence, cancer cell metastasis, and the emergence of drug resistance requires alternative treatment options. The insights from the last several years increasingly support the idea that PDT is a powerful strategy for superficial cancer treatment, such as non-melanoma skin cancer, with advantages of minor damage, few side-effects, and precise treatment. However, some inherent shortcomings limit the clinical application of PDT, such as lack of tumor-specific targeting, penetration depth, and tumor microenvironment properties. Here, we review that PDT alone is not effective enough in some hypoxic and deep solid tumors and may be successfully combined with other therapies to enhance efficacy. Many studies show that the application of nanoparticle-based codelivery methods is very promising and can be expected to speed up the success of PDT combined therapy. In separate delivery, PDT also shows the potential to enhance NP accumulation in tumor areas, which will boost the efficiency of NP-loaded therapeutic agents.

However, as most studies focused on in vitro and in vivo mice models, it is necessary to validate these combination strategies in clinical settings. There are some remaining challenges in the current clinical application of NP-based PDT combination therapy: (1) identification of appropriate targets for the complex tumor environment [247]; (2) improvement of entrapment efficiency, particle stability, and controlled release rate of therapeutic agents from NPs. The accurate drug release control of complex NPs structures is required, which leads to expensive assembly costs for scale-up production and slow approval from FDA; (3) the highly heterogeneous and continuously changing tumor microenvironment is another major challenge that needs to be overcome. Further insights that are needed for the clinical translation of the preclinical studies include (a) optimizing the design (including size, charge, shape, targeted ligands on NPs surface, and stimuli-responsive structure) and synthesis methods (including coprecipitation, inert gas condensation, sputtering, and microemulsion) of nanoparticles that is optimal for the specific cancer therapy [248,249,250], (b) adjusting the targeting and pharmacokinetic behaviors of nanomaterials, in order to improve their safety and efficacy [247], (c) developing more combination strategies to establish precise and personalized treatment, such as the combination of PDT with starvation therapy, gas therapy (nitric oxide (NO)), laser-induced hyperthermia, and ultrasound therapy [251,252,253,254]; and (d) exploring the mechanisms behind the different combinations, in order to control potential side-effects. These insights will provide new ground for transiting NP-based PDT combination therapies to future clinical practice. By overcoming these challenges, PDT combination therapy supported by nanotechnology will become a promising cancer treatment strategy and improve clinical benefit for cancer patients.

## Figures and Tables

**Table 1:** Clinical trials of photodynamic therapy-based combination strategies.

Phase	Photosensitizer	Combined Interventions	Cancer Type	Status	Years of Study	Clinical Trial Reference Number
Phase I	Temoporfin (Foscan^®^)	Surgery	Non-small-cell lung cancer	Completed	2013–2019	NCT01854684
	HPPH (Photochlor^®^)	Surgery	Head and neck cancer	Completed	2007–2018	NCT00470496
	HPPH (Photochlor^®^)	Surgery (laser therapy)	Primary or invasive larynx cancer	Completed	2008–2018	NCT00675233
	Motexafin lutetium	Surgery	Cervical intraepithelial neoplasia	Terminated	2003–2013	NCT00005808
	- (Not marked)	Surgery and radiosensitizer (etanidazole)	Intraperitoneal or pleural cancer	Terminated	2003–2013	NCT00028782
	Porfimer sodium (Photofrin^®^)	Surgery	Malignant mesothelioma	Completed	2003–2011	NCT00054002
	Hematoporphyrin derivative	Radiotherapy (brachytherapy)	Lung cancer	Completed	2004–2013	NCT00014066
	Hexaminolevulinate (HAL)	Placebo ointment	Cervical intraepithelial neoplasia	Completed	2010–2016	NCT01256424
	Aminolaevulinic acid (ALA)	Adjuvant (vitamin D_3_)	Pre-malignant anal tumor	Recruiting	2016–	NCT02698293
	Porfimer sodium (Photofrin^®^)	Chemotherapy (gemcitabine hydrochloride)	Advanced pancreatic cancer	Completed	2013–2018	NCT01770132
Phase II	Aminolaevulinic acid (ALA)	Surgery	Superficial non-melanoma skin cancer	Completed	2003–2013	NCT00002963
	Porfimer sodium (Photofrin^®^)	Surgery and chemotherapy	Non-small-cell lung cancer	Terminated	2008–2020	NCT00601848
	Porfimer sodium (Photofrin^®^)	Surgery and chemotherapy (cisplatin)	Malignant pleural mesothelioma	Completed	2016–2018	NCT02662504
	Porfimer sodium (Photofrin^®^)	Surgery and chemotherapy	Malignant pleural mesothelioma	Recruiting	2014–	NCT02153229
	Hexaminolevulinate (HAL)	Placebo	Cervical intraepithelial neoplasia	Terminated	2008–2013	NCT00708942
	Aminolaevulinic acid (ALA)	Placebo	Cervical intraepithelial neoplasia	Completed	2015–2019	NCT02631863
Phase II/III	Methyl-5-aminolevulinate hydrochloride (Metvix^®^)	Surgery (Ablative CO_2_ laser)	Basal cell carcinoma	Completed	2010–2015	NCT01260987
Phase III	Porfimer sodium (Photofrin^®^)	Chemotherapy (gemcitabine/cisplatin)	Cholangiocarcinoma	Terminated	2014–2019	NCT02082522
	Porfimer sodium (Photofrin^®^)	Chemotherapy (S-1)	Cholangiocarcinoma	Completed	2009–2014	NCT00869635
	Methyl-5-aminolevulinate hydrochloride (Metvix^®^)	Placebo cream	Basal cell carcinoma	Completed	2007–2010	NCT00472108
	Methyl-5-aminolevulinate hydrochloride (Metvix^®^)	Cryotherapy	Basal cell carcinoma	Completed	2007–2010	NCT00469417

**Table 4:** Preclinical studies on PDT plus immunotherapy.

PS	Therapeutic Agents	Delivery System	Therapeutic Outcomes of Combination	Cancer Models	Cytokines	Immune Cells	Ref
BPD-MA	Anti-PD1 post NP-based PDT	Poly (ethylene glycol)-modified metal–organic nanoparticles	Enhanced antitumor efficacy for primary tumor; inhibitory effects on lung metastasis	4T1 murine breast cancer cells	ND	CD8^+^ T cells	[193]
Ce6	Codelivery with DOX to generate in situ Vaccine	Cancer cell membrane (CCM)-coated calcium carbonate (CC) nanoparticles	Enhanced ICD; effective inhibition of both primary and distant growth with low-dose PDT and chemotherapy	4T1 murine breast tumor model	IL-6, IL-12, TNF-α	ND	[194]
	In situ vaccine	Lipid (Li)-coated calcium carbonate (CC) vehicle (Li/CC)	Enhanced inhibitory effects on primary and distant tumor growth	Colorectal cancer	-	-	[195]
	Autologous tumor cell-based vaccines	Fmoc-KCRGDK-phenylboronic acid (FK-PBA) hydrogel	Efficiently inhibited tumor relapse	B16-OVA, CT26	TNF-α, IFN-γ	DCs, Treg CD4^+^/CD8^+^ T cells	[196]
	Codelivery with CpG ODNs to generate in situ vaccine	Mesoporous silica nanoparticles	Enhanced immunogenic cell death; effective accumulation of bMSN in tumors (up to 9.0% ID/g) after intravenous administration; enhanced antitumor efficacy against locally treated tumors and distant, untreated tumors	MC-38 murine colorectal tumor model, B16F10 murine tumor model	IFN-γ	CD8^+^ T cells, DCs	[197]
	In situ vaccine and further anti-PD1 treatment	PDA@UCNP-PEG/Ce6	Strong antitumor immune responses; enhanced antitumor efficacy for primary tumor; inhibitory effects on disseminated tumor growth; inhibitory effects on tumor relapse and metastasis	B16F10c, 4T1 murine tumor model	ND	DCs, CD4^+^/CD8^+^ T cells, memory T cells	[198]
	Codelivery with R837 to generate in situ vaccine and then anti-CTLA4 treatment	UCNP-Ce6-R837 nanoparticles	Strong antitumor immune responses; enhanced antitumor efficacy for primary tumor; inhibitory effects on distant tumor growth; prevented tumor recurrence through a long-term immune memory function	CT26 murine colorectal tumor model	IL-12, IFN-γ, TNF-α	DCs, CD4^+^/CD8^+^ T cells, memory T cells	[199]
	Anti-CTLA4 treatment post NP-based PDT	CM@M-MON@Ce6 nanoparticles	Enhanced ICD; notable eradication of primary and deeply metastatic tumors	MCF-7 murine breast tumor model	TNF-α, IFN-γ, IL-6	DCs, CD4^+^/CD8^+^ T cells, CTLs	[200]
	Codelivery with R837 to generate In situ vaccine and then anti-CTLA4 treatment	Ce6-CAT/PEGDA hybrid hydrogel	Enhanced antitumor efficacy by means of one injection followed by repeated stimulations; inhibitory effects on distant tumor growth; prevented tumor recurrence through a long-term immune memory function	4T1 murine breast tumor model	IFN-γ, TNF-α	DCs, CD4^+^/CD8^+^ T-cells, memory T cells, Tregs, myeloid-derived suppressor cells	[201]
	Anti-PD1 treatment post NP-based PDT	PDA@UCNP-PEG/Ce6	Strong antitumor immune responses; enhanced antitumor efficacy for primary tumor; inhibitory effects on disseminated tumor growth; inhibitory effects on tumor relapse and metastasis	B16F10c, 4T1 murine breast tumor mice model	ND	DCs, CD4^+^/CD8^+^ T cells, memory T cells	[198]
	Anti-PDL1 treatment post NP-based PDT	H-MnO_2_-PEG/C&D nanoparticles	Strong antitumor immune responses; enhanced combating effects of the primary tumor progression; inhibitory effects on untreated distant tumors	4T1 murine breast tumor model	IL12, IFN-γ, TNF-α	Macrophage, cytotoxic T lymphocytes	[202]
	Anti-PDL1 treatment post NP-based PDT	Ce6/MLT@SAB nanoparticles	Improved levels of ICD and abilities to activate dendritic cells in vitro; enhanced PDT killing efficiency in vitro by NPs; augmented antitumor effects	4T1 murine breast tumor model	ND	DCs, CD4^+^/CD8^+^ T cells, myeloid-derived suppressor cells	[203]
	Codelivery with DOX and then treatment with anti-PDL1	Hybrid TKHNP-C/D nanoparticles	Evoked anticancer immune responses; enhanced inhibition of primary and distant tumor growth	4T1 murine breast tumor model	TNF-α, IFN-γ	DCs, CD8^+^ T cells, CTLs	[204]
Cu-doped carbon dots (CDs)	Anti-PDL1 therapy and starving-like therapy after NP-based PDT	γ-PGA@GOx@Mn, Cu-CDs nanoparticles	Improved treatment efficiency; inhibitory effects on nonirradiated tumors due to systematic antitumor immune response	4T1 murine breast tumor model	IFN-γ	CTLs, DCs	[205]
HPPH	Codelivery with Dox to generate in situ vaccine	Chimeric crosslinked polymersomes	Enhanced immunogenic cell death; increased mature DCs in tumor-draining lymph nodes (tdLNs) and CD8^+^ T cells in tumor tissues; enhanced inhibitory effects on primary and distant tumor growth	MC38 murine colorectal tumor model	IL6	CD8^+^ T-cells, DCs	[206]
	In situ vaccine	Graphene (HPPH)–PEGylated GO NPs conjugated with an HK peptide	Effectively ablated primary tumors and destroyed residual tumor cells with SPECT/CT imaging capability; enhanced antitumor immunity and immune memory, which help to prevent distant lung metastasis	4T1 murine breast tumor model	IFN-γ	CD8^+^ T cells, DCs	[207]
H2TCPP	Codelivery with CpG ODNs; in situ vaccine	PCN–ACF–CpG@HA metal–organic nanoparticles	Enhanced immunogenic cell death; effective inhibition of both primary and HIF-1α-induced survival and metastasis	H22 murine hepatic carcinoma cells	TNF-α, IFN-γ, IL-12	DCs	[208]
ICG	Codelivery with siRNA PD-L1	Mn@CaCO_3_/ICG nanoparticles	Efficient delivery of the loaded drug to the tumor tissues; improved tumor hypoxia; roused the immune system	Lewis lung tumor cells	TNF-γ, INF-γ, IL-12, IL-18	DCs, CD4^+^/CD8^+^ T cells	[209]
	Codelivery with R837 and then treat with anti- CTLA4	PLGA-ICG-R837 nanoparticles	Generated more tumor-associated antigens; generated immunological responses will be able to attack remaining tumor cells in mice, which is useful in metastasis inhibition	4T1 murine breast tumor model, CT26 murine colorectal tumor model	IL-12, IL-1β, IL-6, TNF-α, IFN-γ	DCs, CD4^+^/CD8^+^ T cells, memory T cells	[210]
Porphyrin	Codelivery with cetuximab, further treatment with anti-PDL1	EGFR–CPIG liposomal nanohybrid cerasomes	Enhanced antitumor efficacy	CT26 murine colorectal cancer	-	-	[211]
PpIX	Codelivery with CpG ODNs and then anti-PD-L1 therapy	Cu9S5@mSiO_2_-PpIX@MnO_2_ (CSPM) nanoparticles	Notable eradication of primary tumor; Further combined with PD-L1 blockade therapy, showed potential to inhibit metastasis of tumors	4T1 murine breast tumor model	TNF-α, IFN-γ, IL-12	CD8^+^ T-cells, CTLs	[212]
Pyropheophorbide	Codelivery with oxaliplatin to generate in situ vaccine, then combined with anti-PDL1	NCP@pyrolipid core-shell nanoparticles	Enhanced immunogenic cell death and immunity of PDT; regression of primary tumors and distant tumors in bilateral syngeneic mouse	CT26 and MC38 murine colorectal tumor models	IFN-γ, TNF-α	CD4^+^/CD8^+^ T cells	[213]
Pyrolipid	Anti-PDL1 treatment after NP-based PDT	ZnP@pyro nanoparticle	NP-PDT sensitized tumors to checkpoint blockade therapy; enhanced inhibition of primary tumor growth and untreated distant tumors; prevented metastasis to the lung	4T1 murine breast tumor model	IL-6, IFN-γ, TNF-α	Macrophages, DCs	[214]
ZnPc	Codelivery with CpG ODNs	CpG–ODN–Au–ZnPc–poly gold nanoparticles	Increased toxicity of NP-combined therapy than single treatment in vitro; enhanced cytokine levels	4T1 murine breast cancer cells	IL-2, IL-4, IL-6, IL-10, IL-12, TNF-α, IFN-γ	DCs	[215]
Sinoporphyrin sodium (DVDMS)	Codelivery with PD-1 protein by coating onto NP surface (substituting for Anti-PD1)	Human serum albumin (HSA)–perfluorotributylamine @HSA–DVDMS@PD-1 membrane, PHD@PM	Enhanced antitumor efficacy (maturation of DCs and tumor infiltration of CTLs)	4T1 murine breast tumor model	TNFαIL10	DCs, CTLs, Th cells, Tregs	[216]
5,10,15,20-Tetra-(4-aminophenyl) porphyrin	Anti-PDL1 treatment post NP-based chemo-PDT	Copper-doped nanoscale covalent organic polymer	Inhibited tumor growth and activated immune responses; suppressed distant tumor growth and cancer metastasis	CT26 murine colorectal tumor models	INF -γ, TNF-α	DCs, CTLs, CD4^+^/CD8^+^ T-cells	[217]

## Data Availability

Not applicable.

## Abbreviations

The following abbreviations are used in this manuscript: photodynamic therapy (PDT), photosensitizer (PS), singlet or triplet state photosensitizer (PS*), water (H_2_O), triplet oxygen (^3^O_2_), singlet oxygen (^1^O_2_), reactive oxygen species (ROS), hydrogen peroxide (H_2_O_2_), superoxide anions (O_2_^−^) and hydroxyl radicals (OH^−^), basal cell carcinoma (BCC), extramammary Paget’s disease (EMPD), radiotherapy (RT), nanoparticles (NPs), triapazamine (TPZ), apaziquone (EQ4), anoxantrone (AQ4N), glutathione (GSH), aggregation-caused quenching (ACQ), polymeric nanoparticles (PNPs), nanostructured lipid carriers (NLCs), solid lipid nanoparticles (SLNs), gold nanoparticles (AuNPs), up-conversion nanoparticles (UCNPs), conjugated polymer (CP), positron emission tomography (PET), hafnium (Hf; 4+), tetrakis(4-carboxyphenyl) porphyrin (TCPP), enhanced permeability and retention (EPR), arginylglycylaspartic acid (RGD), iodine-125 (^125^I)**,** dibenzocyclooctyne (DBCO), coordination polymer nanoparticles (CPNs), hematoxylin and eosin (H&E), aggregation-induced emission (AIE), photoacoustic (PA) imaging, damage-associated molecular patterns (DAMPs), T helper cells (CD4^+^ T cells), cytotoxic T cell (CD8^+^ T cells), indoleamine 2,3-dioxygenase (IDO), vascular endothelial growth (VEGF), cyclooxygenase (COX)-2, immunogenic cell death (ICD), interferon (IFN), multidrug resistance (MDR), folic acid (FA), hyaluronic acid (HA), polyethylene glycol (PEG), doxorubicin (DOX), double-stranded RNA (dsRNA), myeloid-derived suppressor cells (MDSC), polyglycolic acid (PLA), poly(lactic-co-glycolic acid) (PLGA), cancer stem cells (CSCs), docetaxel (DTX), meso-tetraphenyl chlorine disulfonate (TPCS2a), verteporfin (VP), platinum (Pt), indocyanine green (ICG), florescent imaging (FL), computed tomography (CT), camptothecin (CPT), oxaliplatin (OXP), anthracycline doxorubicin (BDOX), cisplatin (CDDP), mitoxantrone (MX), polydopamine (PDA), bromoisophosphoramide mustard intermediate (IPM-Br), silica (SiO_2_), titanium oxide (TiO_2_), calcium carbonate (CaCO_3_), triple-negative breast cancer (TNBC), chlorambucil (CBL), US Food and Drug Administration (FDA), Toll-like receptors (TLRs), regulatory T cells (Tregs), programmed cell death protein 1 (PD-1), programmed death ligand 1 (PD-L1), cytotoxic T-lymphocyte-associated protein 4 (CTLA-4), cytotoxic T lymphocytes (CTLs), tumor necrosis factor (TNF-α), interleukin 6/10/12 (IL-6/10/12), cancer cell membrane (CCM), dendritic cells (DCs), tumor-associated antigens (TAA), antigen-presenting cells (APCs), synthetic long peptides (SLP), oligodeoxynucleotides (ODNs), collagenase (Col), extracellular matrix (ECM), granulocyte-macrophage colony-stimulating factor (GM-CSF), magnetic resonance (MRI), superparamagnetic NPs (SPIONs), graphene oxide (GO), carbon nanodots (CDs).
